# HBV polymerase overexpression due to large core gene deletion enhances hepatoma cell growth by binding inhibition of microRNA-100

**DOI:** 10.18632/oncotarget.7021

**Published:** 2016-01-25

**Authors:** Ya-Hui Huang, Ying-Hsin Tseng, Wey-Ran Lin, George Hung, Tse-Ching Chen, Tong-Hong Wang, Wei-Chen Lee, Chau-Ting Yeh

**Affiliations:** ^1^ Liver Research Center, Chang Gung Memorial Hospital, Linkou, Taoyuan, Taiwan; ^2^ Molecular Medicine Research Center, Chang Gung University, Taoyuan, Taiwan; ^3^ Department of Hepato-Gastroenterology, Chang Gung Memorial Hospital, Taoyuan, Taiwan; ^4^ Department of Molecular Biology, Princeton University, NJ, USA; ^5^ Department of Pathology, Chang Gung Memorial Hospital, Taoyuan, Taiwan; ^6^ Division of Liver and Transplantation Surgery, Department of General Surgery, Chang Gung Memorial Hospital, Taoyuan, Taiwan

**Keywords:** hepatitis B, hepatocellular carcinoma, molecular oncology, mutation screening, microRNA-100

## Abstract

Different types of hepatitis B virus (HBV) core gene deletion mutants were identified in chronic hepatitis B patients. However, their clinical roles in different stages of natural chronic HBV infection remained unclear. To address this issue, HBV core genes were sequenced in three gender- and age-matched patient groups diagnosed as chronic hepatitis, cirrhosis and hepatocellular carcinoma (HCC), respectively. Functional analysis of the identified mutants was performed. A novel type of large-fragment core gene deletion (LFCD) was identified exclusively in HCC patients and significantly associated with unfavorable postoperative survival. The presence of LFCDs resulted in generation of precore-polymerase fusion protein or brought the polymerase reading frame under direct control of HBV precore/core promoter, leading to its over-expression. Enhanced cell proliferation and increased tumorigenicity in nude mice were found in hepatoma cells expressing LFCDs. Because of the epsilon-binding ability of HBV polymerase, we hypothesized that the over-expressed polymerase carrying aberrant amino-terminal sequence could bind to cellular microRNAs. Screening of a panel of microRNAs revealed physical association of a precore-polymerase fusion protein with microRNA-100. A binding inhibition effect on microRNA-100 by the precore-polymerase fusion protein with up-regulation of its target, polo-like kinase 1 (PLK1), was discovered. The binding inhibition and growth promoting effects could be reversed by overexpressing microRNA-100. Together, HCC patients carrying hepatitis B large-fragment core gene deletion mutants had an unfavorable postoperative prognosis. The growth promoting effect was partly due to polymerase overexpression, leading to binding inhibition of microRNA-100 and up-regulation of PLK1.

## INTRODUCTION

Over 350 million people worldwide suffer from chronic hepatitis B virus (HBV) infection, resulting in about one million deaths per year due to acute liver failure, decompensated liver cirrhosis, and hepatocellular carcinoma (HCC) [1]. During acute and chronic infection, genomic mutations develop in HBV viral sequences. Several of these mutations have been extensively studied, including precore-stop-codon, pre-S deletion, X truncation and basal core promoter (BCP) mutations. However, there remain some mutations, which are less well characterized. Distinct types of naturally occurring HBV core gene deletion mutants have been reported in patients with chronic HBV infection [2-9]. Persistence of such mutants may contribute to the chronicity of diseases [2]. Previous studies showed that these mutants could develop in some special patient populations. For example, core gene deletion mutants accumulated in kidney transplantation recipients and their persistence led to accelerated liver disease progression [4]. A -1G precore/core deletion mutation emerged in human immunodeficiency virus/HBV co-infected patients, which was associated with high serum HBV DNA concentrations [7]. HBV core gene deletion mutants were also found in children younger than 5 years of age, who acquired HBV through horizontal transmission [6]. Despite these studies, the prevalence and clinical roles of core gene deletions in different stages of chronic HBV infected adults have never been carefully investigated.

The HBV core gene encodes a 25-kDa precore precursor protein and a 21-kDa core protein with overlapping reading frames. The signal peptide at the amino-terminus of precore protein is cleaved off and the carboxyl terminal portion is removed to generate a 17-kDa product, named HBV e antigen (HBeAg). The secreted HBeAg accumulates in serum to modulate the immune response to the nucleocapsid [10]. The core protein assembles into core particles. It contains nuclear localization and nuclear export signals, which assist HBV DNA shuttling between nucleus and cytoplasm [11]. Additionally, the core protein is highly immunogenic during chronic HBV infection [12]. The deletion variants of core gene themselves were defective for HBV replication [9] and a longitudinal study from chronic hepatitis B patients receiving interferon-alpha treatment suggested that the core gene deletions might inhibit HBV replication [13]. Mosaic core particles were found when co-expressing wild type and deletion variants [14]. A novel human protein is interacting with only the core deletion mutant but not the wild type [15]. Finally, emergence of core gene deletion variants was likely due to enhanced replication mediated at the level of encapsidation or reverse transcription [16].

In the first stage of this study, we analyzed HBV core gene sequences in three sex- and age-matched chronic hepatitis B patients diagnosed as chronic hepatitis, cirrhosis and HCC to search for core gene deletion mutations. Strikingly, a novel type of large-fragment core gene deletions (LFCDs) was found exclusively in the HCC patients. In such mutants, > 3/4 of the whole core coding region was deleted. To understand whether this type of mutants was associated with hepatocarcinogenesis, a cohort of 179 HBV-related HCC patients receiving surgical removal of HCCs were included for survival analysis. A novel oncogenic mechanism involving physical interaction between HBV polymerase and microRNAs (miRNAs) was proposed and examined in cell-based experiments as well as xenograft mice models.

## RESULTS

### Identification of LFCDs in HCC patients

To investigate the prevalence of core gene deletions in the natural courses of adult chronic hepatitis B patients, the coding regions of precore/core genes including part of the X and polymerase gene (nt. 1557 to 2542) were amplified from the serum samples of chronic hepatitis B patients for sequence analysis. Three groups of patients diagnosed as chronic hepatitis, cirrhosis and HCC were included. The G1896A and BCP mutations data were listed and compared in Table S1. Genotype C was found in 20/33 (60.6%) of HCC patients and in 16/66 (24.2%) of non-HCC patients (*P* = 0.002).

Sequence analysis (reference sequence: GenBank Accession No: NC_003977) revealed 115 core gene nucleotide variations scattered in the three groups. Of these mutations, 26 were different among the three groups (*P* < 0.05). Ten mutations were genotype-associated. The clinical associations of these nucleotide changes were likely due to genotype C association with HCC. Two substitutions at A2144 and C2304 were related to C2 and B4 subgenotype, respectively. The remaining 14 mutations were not related to genotype difference (Table S2). A core internal deletion (CID), which was < 120 nucleotides in length, was found in two cirrhosis patients.

**Figure 1:**
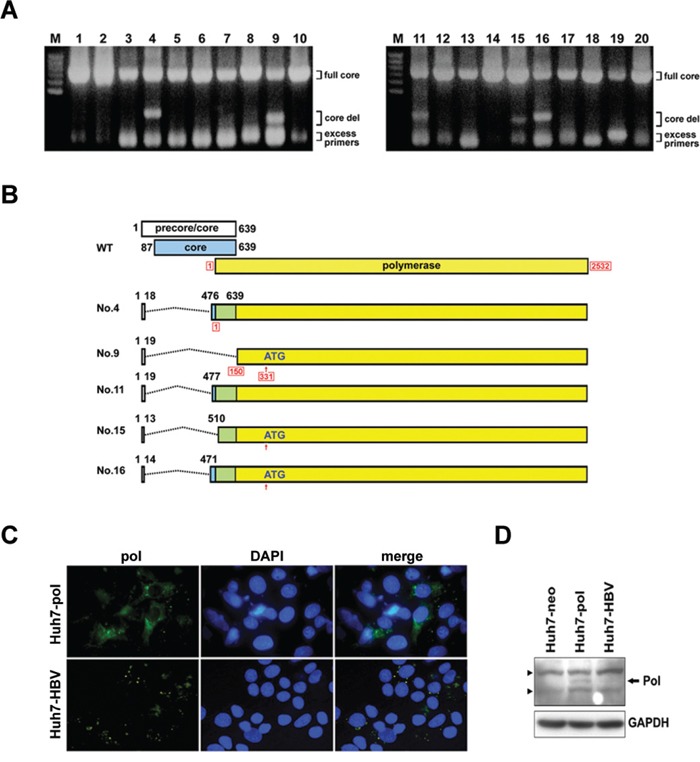
Identification of LFCDs in HCC patients and subcellular localization of HBV polymerase **A.** PCR amplification of the precore/core sequence (nt. 1557 to 2542) of HBV genome. DNA was extracted from the serum samples of HCC patients. The PCR products were analyzed on an agarose gel. The positions of amplicons representing the full length core gene (full core) and deletion mutants (core del) were marked. **B.** The genomic structures of the wild-type (WT) and 5 precore/core gene deletions derived from 5 HCC patients (Patients No. 4, 9, 11, 15 and 16). The dash lines indicated the deleted regions. The nucleotide numbers of precore/core gene were given in black letters. The nucleotide numbers of polymerase gene were given in red letters with frames. **C.** Immunoflouresence analysis for HBV polymerase in Huh7-pol and Huh7-HBV cells. Nuclei were counterstained with DAPI. **D.** Immunoblot analysis for HBV polymerase expression (arrow). Solid triangles indicated non-specific bands. GAPDH served as a loading control.

A novel LFCD, which was > 450 nucleotides in length, was found in the HCC patients (5/33, 15.2%), but not in the other two groups. These 5 LFCDs revealed HBV polymerase mutant with aberrant amino-terminal sequence (pol-N-mut). Among them, the entire polymerase reading frame was intact in the clones derived from patient −4, 11 and 16 (Figures 1A, 1B and Supplementary information). The clones derived from patient −4 and 11 encoded a precore-polymerase fusion protein (preC-pol) containing only 6-7 amino acid residues of the precore amino-terminal sequence, while the patient −16 derived clone had an out-of-frame deletion. In the patient −9 and 15 derived clones, the first encountered ATG was located at nt. 331 of the HBV polymerase gene, which was an internal polymerase ATG (Figure 1B).

Longitudinal serum samples of these 5 patients were retrieved from the serum bank for LFCD detection. It was found that the same LFCDs were maintained after HCC had developed in all patients. In patient −4 and 11, the same LFCDs could be detected 1 month before HCC development. Otherwise, no LFCD was detectable before diagnosis of HCC.

### Overexpression of an HBV preC-pol in the LFCD mutant

To investigate the function of preC-pol, a representative mutant sequence derived from patient −11 was cloned and the sequence carrying the LFCD was used to replace the corresponding coding region in pCMV-HBV [17]. The resulting plasmid, named pCMV-pol, was transfected into Huh7 cells and polymerase expression was detected by immunofluorescence. The pCMV-HBV was also transfected into Huh7 cells for comparison. In pCMV-pol transfected cells, the HBV preC-pol was distributed diffusely in the cytoplasm, whereas in pCMV-HBV transfected cells, only a punctate distribution of polymerase signal was observed (Figure 1C). Immunoblot analysis showed a higher expression level of preC-pol in pCMV-pol transfected cells compared with that in pCMV-HBV transfected cells (7 to 15-folds) (Figure 1D). Similar results were found in HepG2 cells (Figure S1).

LFCD mutants driven by native HBV promoter/enhancer also led to over-expression of polymerase protein. The experimental results were provided in the Supplementary information.

### Overexpression of preC-pol promoted hepatoma cell growth

**Figure 2:**
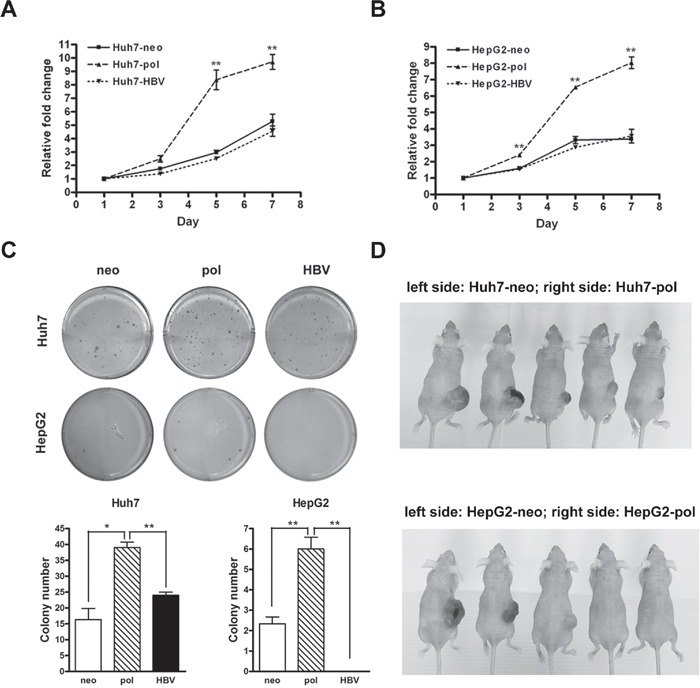
HBV carrying LFCD mutation promoted cell growth *in vitro* and *in vivo* **A** and **B.** Cell proliferation rates assessed by MTT assays for Huh7-neo, -pol, and -HBV cells (A), and for HepG2-neo, -pol, and -HBV cells (B). “**”, *P* < 0.01. **C.** Anchorage independent growth of Huh7 and HepG2 derived stable cell lines in soft agar. Quantitative assessment of the colony numbers for Huh7 (lower left) and HepG2 (lower right) derived stable cell lines. Values were given in mean ± SD from triplicate experiments. “*”, *P* < 0.05; “**”, *P* < 0.01. **D.** Comparison of tumorigenicity in nude mice for Huh7 (upper panel) and HepG2 (lower panel) derived stable transformants. Huh7-neo and HepG2-neo cells were subcutaneously injected into the left side of backs, while Huh7-pol and HepG2-pol cells were injected into the right side of backs for comparison.

Because the LFCD was detected exclusively in HCC patients, we investigated its roles in hepatoma cell growth. The pCMV-pol was transfected into two hepatoma cell lines to generate Huh7-pol and HepG2-pol stable transformants. The empty vector, pRc/CMV, and the wild type control plasmid, pCMV-HBV, were also stably transfected into the two cell lines respectively for comparison. They were named Huh7-neo and HepG2-neo for mock controls, and Huh7-HBV and HepG2-HBV for wild type controls. In MTT assay, it was found that cell proliferation increased significantly in Huh7-pol cells, when compared with that in Huh7-neo or Huh7-HBV cells (Figure 2A). Similar results were also found in HepG2 cells (Figure 2B). In soft agar assay, the colony numbers in Huh7-pol and HepG2-pol cells increased significantly, when compared with those of the other two controls (Figure 2C).

In xenograft models, five-week-old nude mice were subcutaneously injected with Huh7-neo (left side of back) and Huh7-pol (right side of back) cells. No tumor formation was observed in the area where Huh7-neo cells were injected during the observation period (6 wks; n = 10). However, 9 (90%) tumors developed in the corresponding areas injected with Huh7-pol cells (Figure 2D, upper panel). Similar results were found using HepG2-neo and HepG2-pol cells. Of the 10 mice, no HepG2-neo xenograft tumor was found during the observation period (7 wks; left side of back), whereas 6 (60%) tumors developed in HepG2-pol cells injected sites (Figure 2D, lower panel, right side of back). Additionally, no tumor formation occurred in the areas injected with Huh7-HBV or HepG2-HBV cells (n = 10; separate sets of mice in two independent experiments) in the same periods of time (data not shown). Chi-square test analysis indicated the differences between the -pol and -neo groups in Huh7 (*P* < 0.001) and HepG2 (*P* = 0.011) xenograft models.

### PreC-pol overexpression in the LFCD mutants inhibited hepatoma cell apoptosis

**Figure 3:**
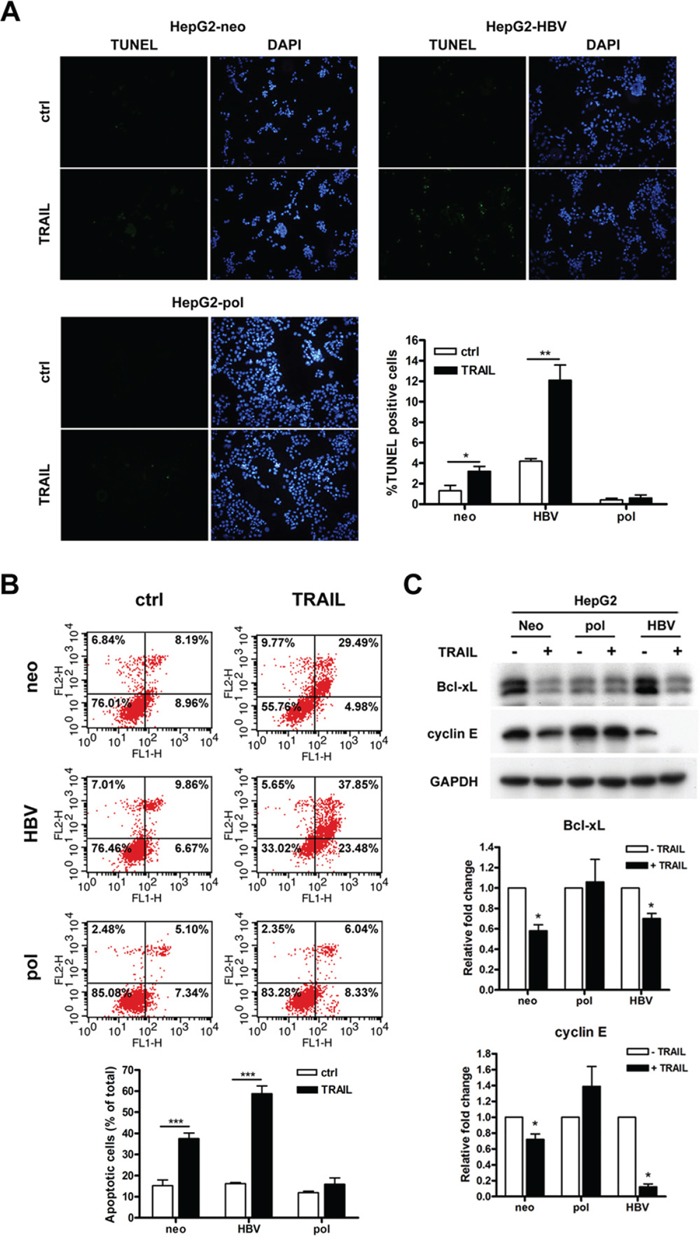
HBV carrying LFCD mutation inhibited TRAIL-induced cell apoptosis in HepG2 cells **A.** TUNEL assays for HepG2-neo, -pol and -HBV cells after they were treated with 100 ng/mL of TRAIL for 24 h. Cells exhibited green fluorescence were under apoptosis. Nuclei were stained with DAPI. Quantitative comparison for the numbers of apoptotic cells were depicted (right lower panel). **B.** After treated with TRAIL, HepG2 stable cells were stained with annexin V-FITC and PI for flow cytometric analysis. Lower panel, quantitative comparison of the numbers of apoptotic cells. **C.** Comparisons of Bcl-xL and cyclin E expression levels after HepG2 cells derived stable transformants (-neo, -pol, and -HBV) were treated with (+) or without (−) TRAIL. GAPDH served as a loading control. Top panel, immunoblot analysis; Middle and bottom panels, quantitative assessments. Values were given in mean ± SD from three independent experiments. “*”, *P* < 0.05; “**”, *P* < 0.01.

Tumor necrosis factor-related apoptosis-inducing ligand (TRAIL) could induce apoptosis in HCC cells [18]. This effect was enhanced when the HCC cells were stably transfected with a HBV genome [19]. To understand the effect of core deletion-associated polymerase overexpression in TRAIL-induced apoptosis, HepG2 and Huh7 cells stably transfected with pRc/CMV (-neo), pCMV-pol and pCMV-HBV, respectively, were treated with TRAIL. By use of terminal deoxynucleotidyl transferase dUTP nick end labeling (TUNEL) assay, apoptotic cells were increased by TRAIL treatment (3.2%) in HepG2-neo cells as compared with untreated control (1.3%) (*P* < 0.05). In consistent with the previous report [19], the enhancement of the percentages of apoptotic cells was found by TRAIL treatment (12.1%) in HepG2-HBV cells as compared with untreated control (4.2%) (*P* < 0.01). However, the percentages of apoptotic cells had no significantly change by TRAIL treatment (0.6%) in HepG2-pol cells as compared with untreated control (0.4%) (Figure 3A). These findings were also supported by FITC-Annexin V and PI staining. The total apoptotic cells were significant increased by TRAIL treatment in HepG2-neo (37.5%) and HepG2-HBV (58.8%) compared to those untreated control (15.2% and 16.2%) (*P* < 0.001). However, the number of total apoptotic cells had no significantly change between HepG2-pol cells with (15.9%) or without (11.9%) TRAIL treatment (Figure 3B). Similar TUNEL assay and FITC-Annexin V staining results were also found in Huh7-neo, -HBV, and -pol cell lines with or without TRAIL treatment (Figures S2A and S2B). In addition, the expression level of Bcl-xL (anti-apoptotic protein) and cyclin E (cell cycle-related protein) was significant decreased in the pRc/CMV (-neo) and pCMV-HBV transfected HepG2 and Huh7 cells after TRAIL treatment. Interestingly, the down-regulation of Bcl-xL and cyclin E by addition of TRAIL was prevented in HepG2-pol and Huh7-pol cells (Figures 3C and S2C).

### HCC patients harboring LFCDs accompanied by HBV polymerase over-expression had unfavorable postoperative survivals

**Figure 4:**
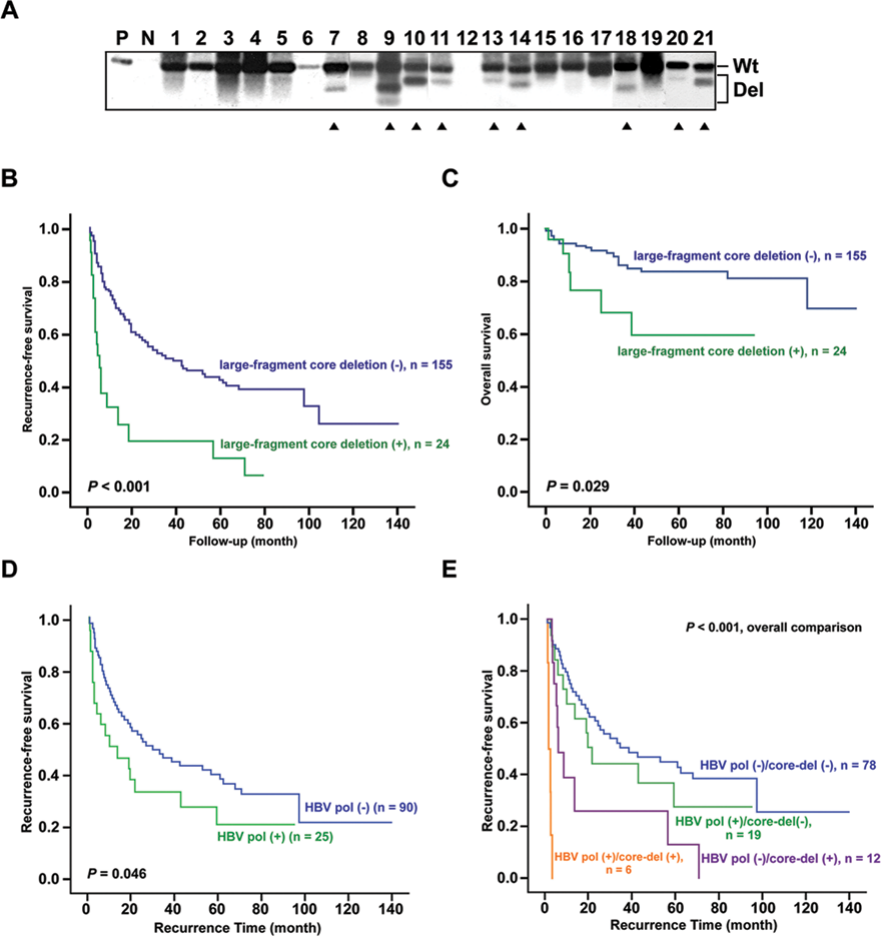
Association between postoperative survivals and HCC patients carrying LFCDs with or without hepatic HBV polymerase antigen expression **A.** Southern blot analysis of precore/core PCR amplicons derived from liver tissues of HCC patients. The positions of PCR products containing wild type (Wt) and LFCD (Del) were marked to the right. P, 1 pg of PCR product from Wt precore/core sequence as positive control; N, 10 pg of non-HBV DNA as negative control; Solid triangles, patients with LFCD. **B** and **C.** Kaplan-Meier survival analysis for postoperative recurrence-free survival (B) and overall survival (C) between HCC patients with (+, green lines) and without (−, blue lines) LFCD. **D** and **E.** Comparison of postoperative recurrence-free survivals between patients with hepatic HBV polymerase antigen positive (+) and those with polymerase antigen negative (−) reaction by immunohistochemistry (D). Combined analysis of polymerase (+)/(−) and LFCD (+)/(−) status (E). Kaplan-Meier analysis was used.

Because the case-control study as well as the cell- and animal-based experiments all suggested that polymerase overexpression due to LFCD was associated with enhanced hepatoma cell growth (Figures 2 and S3), we searched for further clinical relevance in postoperative HCC patients. A cohort of 155 male and 24 female HBV-related HCC patients receiving surgical removal of tumors were included for survival analysis. Of the 179 patients, 117 (65.4%) were genotype B and 62 (34.6%) were genotype C. The basic clinical data stratified by genotypes were listed and compared in Table S3. The LFCD mutants were detected in 13 (11.1%) genotype B and 11 (17.7%) genotype C patients (*P* = 0.215). The core gene was detected by PCR followed by Southern blot analysis. Wild type (wt) and various sizes of LFCD (Del) mutants from HCC patients were found (Figure 4A).

The Cox proportional hazard model was performed to analyze whether these HBV genomic mutations were independent prognostic predictors when clinicopathological variables were included. As shown in Table S4, univariate analysis revealed that ascites, tumor size > 5.5 cm (diameter), microvascular invasion, aspartate transaminases (AST) > 48 U/L, prothrombin time > 12 sec, alpha-fetoprotein (AFP) > 30.5 ng/mL, genotype C, HBV-DNA > 22.67 copies/gram, and three specific mutations (BCP A1762T/G1764A, small-fragment pre-S and LFCD mutations) were significantly associated with recurrence-free survivals. These significant factors were included for multivariate analysis, which showed that ascites, microvascular invasion, BCP A1762T/G1764A and LFCD mutation were the remaining independent factors. In addition, the ascites, tumor size > 5.5 cm (diameter), AST > 48 U/L, BCP A1762T/G1764A and LFCD were significantly associated with overall survivals using univariate analysis. However, in multivariate analysis, only ascites and BCP A1762T/G1764A were independently associated with overall survival. Moreover, HCC patients carrying LFCDs also correlated with a shorter recurrence-free (*P* < 0.001) and overall (*P* = 0.029) survivals by Kaplan-Meier survival analysis (Figures 4B and 4C).

The expression of HBV polymerase in non-cancerous liver tissues from a cohort of 115 HBV-related HCC patients was assessed by immunohistochemistry to understand its correlation with survival. The representative samples of the immunohistochemistry for HBV polymerase (−) and (+) were shown in Figure S4A and S4B. Among them, 25 (21.7%) patients with positive hepatic HBV polymerase staining significantly correlated with a shorter recurrence-free survival (*P* = 0.046) (Figure 4D). When combined with LFCD, the patients without either unfavorable factor had the most favorable postoperative survivals (Figure 4E, blue group). In contrast, 6 patients carried both unfavorable factors had the poorest postoperative survivals (Figure 4E, yellow group). In the remaining 19 polymerase antigen positive patients, LFCD was undetectable possibly due to (i) different origin of tissue polymerase antigen (from integrated HBV genome) or (ii) low concentrations of LFCD under detection limit. To further clarify this issue, we performed immunoblot analysis following immunoprecipitation (IP) for patients who had enough amounts of remaining liver surgical specimens (including the 6 patients with LFCD). As shown in Figure S4C, positive polymerase antigen signals (with various lengths due to deletions) in IHC-positive samples and negative polymerase signals in IHC-negative samples were demonstrated in the immunoblot assay.

### Physical interaction between preC-pol and miRNAs

**Figure 5:**
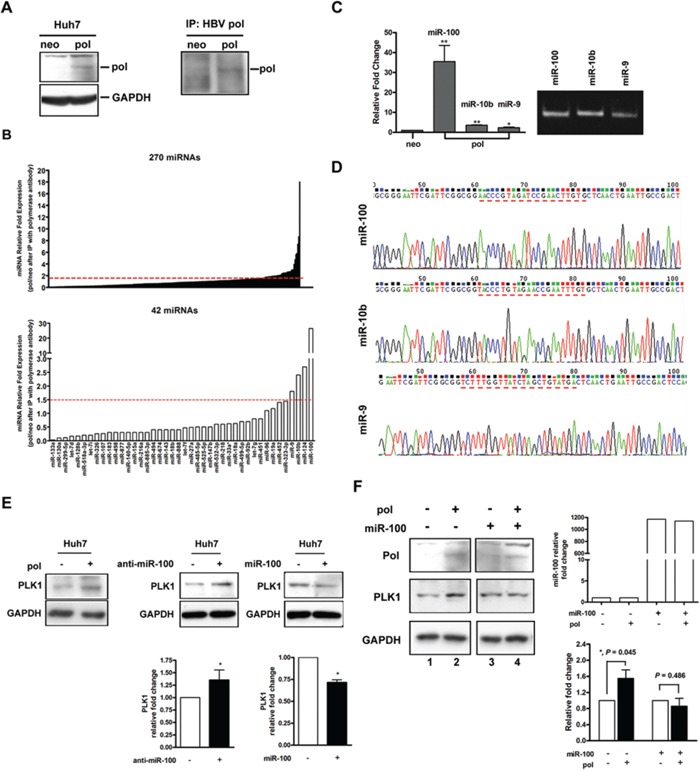
Binding inhibition of miR-100 by HBV preC-pol led to up-regulation of PLK1 **A.** Huh7 cells were transfected with pCMV-pol (pol) or pRc/CMV (neo). HBV polymerase expression was detected by immunoblot analysis (left panel) or immunoprecipitaion using HBV pol antibody followed by immunoblot (right panel). **B.** Screening for microRNAs physically bound to HBV preC-pol. After transfected with pCMV-pol or pRc/CMV, immunoprecipitation was performed using HBV pol antibody. Total RNA was extracted from the immunoprecipitates and submitted for quantitative assessment for a panel of 270 miRNAs (upper panel) and 42 miRNAs (lower panel) by real-time RT-qPCR. **C.** Confirmation of miR-100, miR-10b and miR-9 interact with HBV preC-pol by using individual miRNA primer sets for RT-qPCR. The RT-qPCR products were analyzed on a DNA polyacrylamide gel (right). **D.** Validation of the RT-qPCR products by cloning into a pGEM-T vector for sequence analysis. **E.** PLK1 protein levels in Huh7 cells transfected with (+) or without (−) pCMV-pol (left), anti-miR-100 (middle) or miR-100 (right). Lower panels: quantitative assessments. “*”, *P* < 0.05. **F.** PLK1 protein levels in pCMV-pol transfected or untransfected Huh7 cells with or without overexpression of miR-100. The relative expression level of miR-100 was assessed by RT-qPCR (top right). Quantitative data of PLK1 expression was given (bottom right). Values were given as mean ± SD from three independent experiments. “*”, *P* < 0.05.

The function of HBV polymerase in HBV replication involved recognition and binding to a specific HBV RNA sequence element, called epsilon (ε). This element consisted of a 60 nt-length bulged stem-loop and served as the docking site of polymerase for initiation of reverse transcription [20]. Similarly, miRNAs were processed from a ∼70 nt-length of precursor miRNA (pre-miRNA) folding into a stem-loop structure [21]. In view of the structure similarity between the ε and pre-miRNA, we hypothesized that some pre-miRNAs might interact with HBV polymerase. To investigate this possibility, immunoprecipitation was performed using anti-HBV polymerase antibody for protein extracted from Huh7 cells transfected with or without pCMV-pol (Figure 5A). Total RNA was extracted from the immunoprecipitates to search for polymerase-interacting pre-miRNAs. A panel of 270 miRNAs was selected for subsequent screening experiment because their abundance in liver tissues was high enough for reliable quantification [22]. Pilot screening was performed by multiplex RT-qPCR method using 6 RT primer mixture sets (each set containing 45 miRNA RT primers) to perform stem-loop RT-qPCR. Forty-two miRNAs had a level ≥ 1.5-fold when compared with the mock control (Figure 5B, upper panel). Further quantitative assays focusing on these 42 miRNAs was performed by real-time RT-qPCR using 2 RT primer mixture sets (each set with 27 and 15 miRNA RT primers respectively) after they were brought down by immunoprecipitation. Of them, 4 miRNAs (miR-9, miR-10b, miR-124 and miR-100) remained to have a ≥ 1.5-fold difference (Figure 5B, lower panel). After three steps of screening (Supplementary information), only three of them (miR-100, miR-10b and miR-9) remained significantly enriched in the polymerase immunoprecipitates. The amounts of miR-100, miR-10b and miR-9 in the immunoprecipitates were 35.5-, 3.6- and 2.3-fold enriched, respectively, compared with the mock control (*P* < 0.05) (Figure 5C). To further verify this result, the same experiments were performed in Huh7-neo, Huh7-HBV and Huh7-pol stable transformants. As shown in Figure S5, preC-pol in Huh7-pol stable cell lines still displayed physical interaction with the three miRNAs. However, such interactions were not observed in Huh7-HBV stable cell lines, except for miR-9. The three miRNAs obtained with the immunoprecipitation methods were further submitted for sequence analysis for validation (Figure 5D). The experimental results indicated that there was a physical association between preC-pol and the three miRNAs.

### Up-regulation of polo-like kinase 1 (PLK1) level by overexpression of preC-pol through its interaction with miR-100

Previous report indicated that the PLK1 was a target of miR-100 [23]. Immunoblot analysis showed that the expression level of PLK1 was indeed up-regulated and down-regulated in Huh7 cells upon silencing and overexpressing miR-100, respectively (Figure 5E, middle and right panel). When preC-pol was overexpressed, PLK1 was also up-regulated in Huh7 cells (Figures 5E, left panel and 5F, lane 1 vs. lane 2), mimicking the miR-100 silencing effect. The expression levels of PLK1 also increased in xenografts carrying pHBV1.3-476 and pHBV1.3-477 when compared with those of wild type (pHBV1.3) (Figure S6A). This PLK1 up-regulatory effect was inhibited by overexpression of miR-100 (Figure 5F, lane 3 vs. lane 4 and lower right panel). The expression levels of PLK1 were not significantly different between Huh7 cells with and without pCMV-HBV transfection (Figure S6B). Even when Huh7 cells were simultaneously with pCMV-HBV and miR-100 expressing plasmid, the degree of PLK1 down-regulation was similar to that of miR-100 overexpression alone (Figure S6C, lane 3 vs. lane 4). To further understand the function of miR-100 on hepatoma cells, HepG2 cells, which harbored higher endogenous miR-100 (than did Huh7 cells), were used to knockdown miR-100 for xenograft experiments. As shown in Figure S7, promotion of tumor growth was observed in xenografts with miR-100 down-regulated, when compared to the controls. The tumor promotion effect was similar to but less prominent than that observed in xenografts expressing preC-pol (Figure 2). This result indicated that the physical interaction between preC-pol and miR-100 resulted in an inhibitory effect on the miR-100 function, leading to PLK1 up-regulation.

### Suppression effects of tumorigenicity by miR-100 in Huh7-pol xenografts

**Figure 6:**
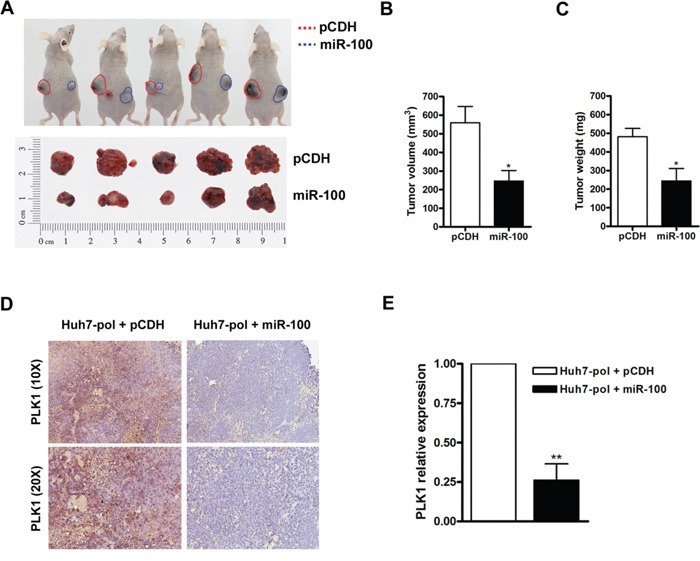
Suppression of tumor growth by miR-100 in Huh7-pol xenografts **A** to **C.** Huh7-pol cells with (blue dash line) or without (red dash line) miR-100 overexpression were subcutaneously injected into nude mice. The dissected xenograft tumors were shown (A, bottom panel). The tumor volume (B) and tumor weight (C) were calculated. Values were mean ± SD. “*”, *P* < 0.05. **D.** The xenograft tumors were subjected for PLK1 detection by use of immunohistochemistry. **E.** The relative expression levels of PLK1 were quantified using Aperio ImageScope system. Values were mean ± SD. “**”, *P* < 0.01.

Subsequently, we examined the regulatory mechanism mediated by preC-pol-miR-100 interaction in the xenograft model. Huh7-pol cells without and with miR-100 overexpression were subcutaneous injected into nude mice on the left (pCDH group) and right (miR-100 group) side of back, respectively. Three weeks later, all mice were sacrificed for measurement of the tumor volume and weight. The levels of miR-100 expressed in Huh7-pol xenografts were assessed using stem-loop RT-qPCR (Figure S8). The final volume and weight of xenografts in the miR-100 group were significantly smaller and lighter than the paired tumors in the pCDH group (Figures 6A to 6C). Decrease of PLK1 expression in the miR-100 group was observed when compared with that in the pCDH group (Figures 6D and 6E). Similarly, significant suppression of cell proliferation was found in Huh7-pol and HepG2-pol cells expressing miR-100 by MTT assay (Figure S9, Day 4, Huh7-pol: 12.3-fold ± 0.42 vs. 9.5-fold ± 0.03; HepG2-pol: 10.0-fold ± 0.19 vs. 7.8-fold ± 0.71). Despite the cell proliferation was also decreased in -neo and -HBV cell lines expressing miR-100, the reduction was less prominent in these two types of cell lines (Figure S9, Day 4, Huh7-neo: 8.4-fold ± 0.48 vs. 6.8-fold ± 0.18; Huh7-HBV: 9.2-fold ± 0.47 vs. 7.8-fold ± 0.80; HepG2-neo: 7.0-fold ± 0.36 vs. 6.4-fold ± 0.49; HepG2-HBV: 6.1-fold ± 0.24 vs. 5.5-fold ± 0.40). P values were given in the figure legends.

### HCC patients with positive HBV polymerase IHC staining accompanied by low miR-100 or high PLK1 expression levels had unfavorable postoperative survivals

**Figure 7:**
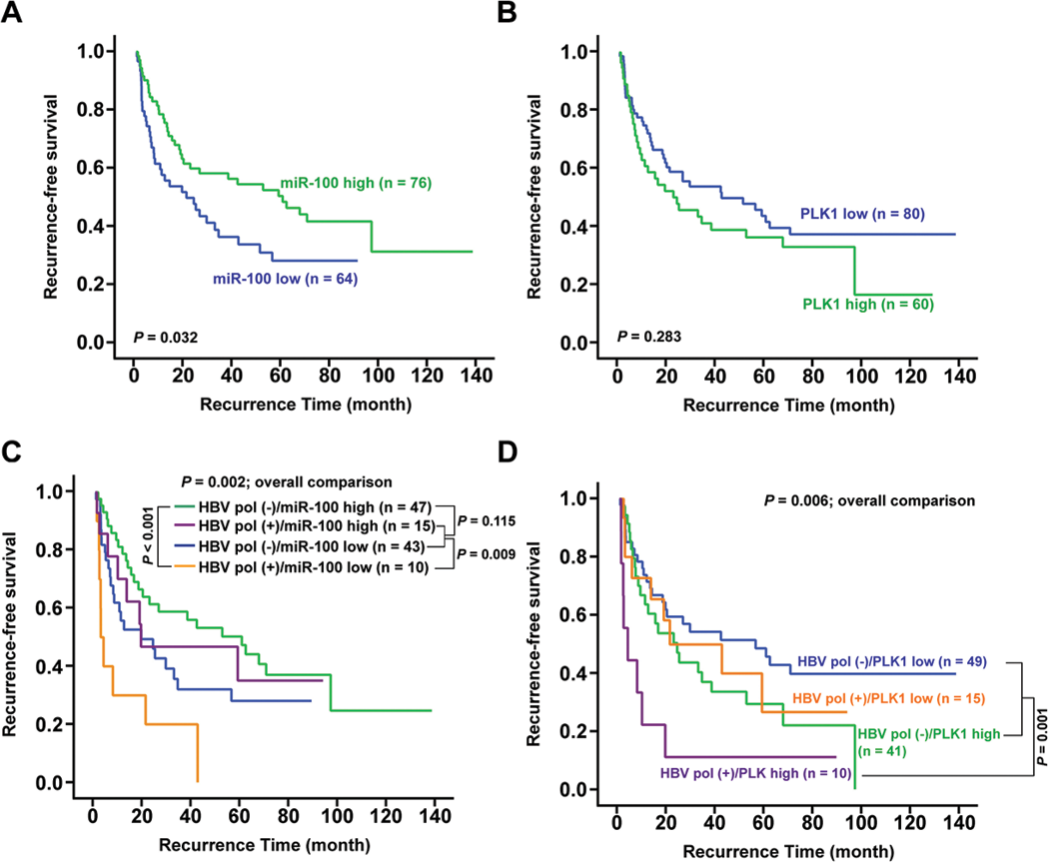
Association between postoperative survival and hepatic miR-100, PLK1 and HBV polymerase antigen expression **A** and **B.** Kaplan-Meier analysis for postoperative recurrence-free survivals between HCC patients expressing high and low hepatic levels of miR-100 (A) or PLK1 (B). **C** and **D.** Combined analysis of postoperative recurrence-free survival. (C), between patients with hepatic HBV polymerase antigen (+)/(−) and miR-100 high/low levels; (D), between patients with hepatic HBV polymerase antigen (+)/(−) and PLK1 high/low levels.

To further investigate the clinical correlation of miR-100 or PLK1 expression in HCC patients, 140 samples derived from non-cancerous liver tissues of HBV-related HCC patients were analyzed. HCC patients with low miR-100 (*P* = 0.032) and high PLK1 (*P* = 0.283) expression level had a shorter recurrence-free survival by Kaplan-Meier survival analysis (Figures 7A and 7B). Interestingly, when combined with HBV polymerase staining results (Figure 4D), patients with both HBV pol (−) and high miR-100 expression had favorable postoperative survival (Figure 7C, green group). In contrast, patients with both HBV pol (+) and low miR-100 expression had the poorest survival (Figure 7C, yellow group; *P* < 0.001). When compared with the purple group (HBV pol (+) and high miR-100 expression) and blue group (HBV pol (−) and low miR-100 expression), the yellow group had significant unfavorable survival (*P* = 0.009). In addition, comparison of the recurrence-free survival in four groups was significant (*P* = 0.002).

As for the combination of PLK1 and HBV polymerase expression, patients with both HBV pol (+) and high PLK1 expression (Figure 7D, purple group) had the poorest survival (*P* = 0.001) when compared with the other three groups (patients with HBV pol (−) and low or high PLK1 expression, or HBV pol (+) and low PLK1 expression) (*P* = 0.006 for overall comparison). Furthermore, among the 115 patients, Pearson's correlation analysis revealed a negative correlation between miR-100 and PLK1 (r = −0.191, *P* = 0.041).

## DISCUSSION

Naturally occurring short internal HBV core gene deletions have been detected in patients with chronic hepatitis B [2, 8]. The large fragment core gene deletions can also be generated through RNA splicing to generate defective HBV genomes [24, 25]. The donor sites of splicing could be located at the carboxyl terminus or at the middle part of core reading frame [26, 27]. By analyzing HBV core gene sequences in the HBV-infected patient groups, we identified two short CID (< 120 nt.) in the cirrhotic patients (2/33, 6.06%) and a novel core gene deletion (LFCD, > 450 nt.) in HCC patients (5/33, 15.1%). Presumably, these deletions could be generated through mis-splicing with a new donor site located at the precore region. A recent longitudinal analysis discovered the HBV splicing events increased prior to development of HCC [26]. This could be the reason why the LFCDs were only found in HCC patients. To date, 14 different HBV splicing variants (Sp1 to Sp14) have been identified [26, 28]. In the precore/core coding area, splicing donor sites were found in 5 of the 14 known variants. None of these variants are the same as LFCD mutants discovered in this study. It can be speculated that there are novel aberrant splicing events. Three patients with LFCD had intact polymerase coding sequence in this study. Of them, two patients had an in-frame precore/core deletions and one patient had an out-of-frame deletion. The other two patients with LFCD had out-of-frame precore/core deletions, resulting in partial deletion of the amino-terminal portion of the polymerase gene (Figure 1). In out-of-frame deletions, an internal start codon located at nt. 331 of the polymerase gene was found. To our knowledge, core gene deletions of such a long fragment have never been reported. The longest documented core gene deletion was 381 nucleotides in length discovered in a patient receiving kidney transplantation [4]. Most core deletions in chronic HBV-infected patients ranged from 24 to 250 nucleotides in length [2, 13, 16].

The BCP, located at nt. 1744 to 1804 of HBV genome, is a major promoter controlling the transcription of the precore/core gene [29]. Following generation of the core-polymerase transcripts, a large amount of the core proteins are translated from the transcripts, whereas only a small amount of polymerase is produced by a ribosomal leaky scanning mechanism [30]. Subsequently, the polymerase together with the pregenomic RNA is immediately encapsidated by the core proteins, allowing for reverse transcription inside the nucleocapsids. As such, few polymerase protein molecules can be detected in the cytosol during normal HBV replication cycles [31, 32]. In LFCD mutants carrying in-frame precore/core deletions (patient −4 and 11), fusion occurred between a short precore sequence (6-7 a.a.) and the polymerase reading frame. As such, the polymerase is no longer generated by the ribosomal leaky scanning mechanism, but is now under direct control of HBV precore/core promoter. Alternatively, in LFCD mutants which out-of-frame deletions (patient −9, 15 and 16), it was likely that translation of these pol-N-mut still relied on the ribosomal scanning mechanism but with the initiation codon far closer to the 5′ end of the transcripts. As a result, the production of pol-N-mut was higher than the wild type (Figure S3C). Furthermore, because of the absence of intact core protein, the polymerase is freely released into cytosol, allowing for its interaction with cytosolic cellular content. At present, the physiological role of the LFCD in HBV life cycle is unknown. However, our results showed that HBV genome carrying LFCD promoted HCC cell growth (Figures 2 and S3), suggesting a survival benefit for HCCs carrying the LFCD mutants. The results were consistent with the postoperative prognostic analysis indicating that HCC patients with LFCDs had shorter recurrence-free and overall survivals (Table S4 and Figures 4B and 4C). When combined with the effect of HBV polymerase, the HCC patients with both LFCD (+) and HBV polymerase (+) exhibited the poorest survival (Figure 4E), suggesting that hepatic HBV polymerase overexpression caused by LFCDs might exert a stronger growth enhancing effect on HCC, when compared with HBV polymerase derived from other possible sources, for example, the aberrant integrated HBV genomes.

Although suppression of TRAIL-induced apoptosis in Huh7-pol and HepG2-pol cells was observed, a lower baseline Bcl-xL level was observed when compared to that of the other two controls (-neo and -HBV) (Figures 3C and S2C). We speculated that it was due to a compensatory regulatory effect. In this view, Huh7-pol and HepG2-pol cells had an increased cell proliferation rate (Figure 2) and therefore an increased cell turnover rate (increased cell necrosis due to environmental limiting factors). As a result, the apoptosis rate was reduced (not needed), accompanied by a decreased Bcl-xL level (anti-apoptosis no longer required). Additionally, cyclin E expression was higher in HepG2-pol cells (Figure 3C) compatible with the increased growth rate in Figure 2B. However, the level was lower in Huh7-pol cells (Figure S2C), suggesting that the increased growth rate in Figure 2A was likely due to further decrease of cell death rate (either from decreased apoptosis or necrosis or other unrecognized causes).

The preC-pol might be able to bind miRNAs was hypothesized because the structural similarity between ε element and miRNAs. Indeed, the miR-9, miR-10b and miR-100 were discovered physically interact with preC-pol. The greatest abundance of miR-100 in the immunoprecipitate implied that polymerase-miR-100 interaction might exert a biological effect (Figure 5C). The miR-100 was able to suppress tumor growth in several cancers, including HCC. Furthermore, it was known that PLK1 was an important target of miR-100 [23, 33]. Previous studies indicated that PLK1 frequently overexpressed in HCC tissues and its overexpression was associated with hepatocarcinogenesis as well as unfavorable clinicopathological outcomes [23, 34, 35]. Our data indicated that preC-pol overexpression in the LFCD mutant could exert an effect mimicking that of anti-miR-100, resulting in PLK1 up-regulation to promote tumor growth. This phenomenon could be reversed by overexpression of miR-100 *in vitro* and *in vivo* (Figures 5F and 6). According to these results, the preC-pol-induced PLK1 overexpression was likely mediated through its binding to miR-100 to inhibit its function. Clinical analysis showed that miR-100 level was associated with postoperative prognosis in HBV-related HCCs. However, when PLK1 was examined, the clinical correlation was not statistically significant. Combined analysis showed that HBV polymerase (+) was modifying the clinical predictive effect of miR-100. Moreover, patients with HBV polymerase (+) and high PLK1 level had the worst postoperative survival, suggesting that HCCs in which miR-100 was greatly inhibited by HBV polymerase overexpression, resulting in higher level of PLK1, had the greatest growth advantage (Figure 7).

PLK1 has been proposed to be a new therapeutic target for HCC recently [36]. Several PLK1 inhibitors such as GSK461364A, BI2536 and ZK-Thiazolidinone were designed to deplete or inhibit PLK1 kinase activities. Clinical trails are undergoing to evaluate their efficacies. Based on our data, HBV-related HCC patients, who have LFCD mutants accompanied by increased PLK1 expression, may be a suitable group for anti-PLK1 therapy.

In summary, a novel type of HBV core gene deletion mutant was identified. The LFCD led to generation of pol-N-mut, resulting in its overexpression and release into cytosol. The cytosolic HBV preC-pol physically interacted with miR-100 to inhibit its normal function, leading to PLK1 overexpresssion, promoting HCC cell growth.

## MATERIALS AND METHODS

### Patients

This study was conducted under the approval of Institutional Review Board, Chang Gung Memorial Hospital. Written informed consent was obtained from all patients. The serum samples from three gender- and age-matched groups diagnosed as chronic hepatitis, cirrhosis, and HCC (n = 33 for each group), respectively, were collected for initial HBV core gene sequence analysis. The basic clinical parameters of these patients were listed in Table S1. Paraneoplastic liver tissues from a cohort of HBV-related HCC patients (155 male and 24 female) were retrieved from Tissue Bank, Chang Gung Medical Center for correlation analysis between postoperative survivals and various clinicopathological/virological factors. In this study, only patients with positive tissue HBV-DNA were included. The baseline characteristics of these 179 patients were listed in Table S3.

### Plasmid construction

The amplicon (nt. 1653 to 2571) carrying LFCD mutation was first cloned into pCRII-TOPO vector (Invitrogen, Carlsbad, CA) and sequence-verified. The *Hind*III (on the vector, next to the 5′end of insert) to *Bgl*II (nt. 2399) DNA fragment carrying the LFCD was isolated from the plasmid and used to replace the *Hind*III (also on the vector, next to the 5′ end of inserted HBV genome) to *Bgl*II (nt. 2399) DNA fragment in pCMV-HBV, a plasmid carrying a 3.37 kb, greater-than-unit-length HBV genome [17]. The resulting plasmid was named pCMV-pol, which contained the LFCD mutation and the whole remaining part of HBV genome.

### Cell culture and stable cell line generation

Huh7 and HepG2 cells were transfected with pRc/CMV (Invitrogen, Carlsbad, CA), pCMV-pol or pCMV-HBV using TransIT-LT1 reagent (Mirus, Madison, WI) to generate Huh7-neo, Huh7-pol, Huh7-HBV, HepG2-neo, HepG2-pol and HepG2-HBV cell lines. The culture medium of transformants was collected for HBsAg measurement to confirm the success of stable transfection.

### Immunofluorescence

Cells were grown on cover slip, fixed in acetone and stained with Hep B pol (2C8) antibody (Santa Cruz Biotechnology, Santa Cruz, CA) followed by anti-mouse IgG-FITC antibody (Leinco Technologies, Ballwin, MO). Cell nuclei were visualized by DAPI staining.

### Assessment of cell growth and apoptosis

Cell proliferation and TUNEL assay were carried out as described [22].

### Xenograft models

All animal experiments were conducted under the approval of Chang Gung Institutional Animal Care and Use Committee. Three-week-old BALB/c nude mice purchased from the National Laboratory Animal Center (Taipei, Taiwan). The procedure was performed as described previously [22].

### Lentiviral transduction

Lentiviral transduction assay was carried out as described [22]. The pCDH-CMV-MCS-EF1-copGFP (pCDH), pmiRZip, pCDH-miR-100 and pmiRZip-100 plasmids were purchased from SBI System Biosciences (Mountain View, CA).

### Immunoprecipitation analysis

Protein lysate from Huh7 cells transfected with pRc/CMV or pCMV-pol was reacted with solution containing protein A/G beads (Santa Cruz Biotechnology), M-PER reagent (Thermo Scientific, Pittsburgh, PA), proteinase inhibitor (Thermo Scientific), RNase inhibitor (H T Biotechnology Lid., Cambridge, UK) and Hep B pol antibody. The immune complex beads were submitted for total RNA extraction using TRIzol reagent (Invitrogen) for miRNA detection.

### MiRNA detection

A stem-loop RT-qPCR method was carried out as described [22].

### Statistical analysis

One-way ANOVA and Chi-Square test were conducted to compare the differences of parametric and dichotomous data, respectively, among patients with chronic hepatitis, cirrhosis and HCC. Post hoc analysis was performed to examine the statistical significance between two selected groups. Student's t-test, Mann-Whitney test and Pearson's correlation analysis were applied, where they were appropriate. In the postoperative survival analysis, associations between the survivals (recurrence-free survival and overall survival) of patients with and without LFCD were calculated using univariate and multivariate Cox regression and confirmed by Kaplan-Meier method. Data were statistically significant when *P* < 0.05. Statistical analysis was conducted by using SPSS version 12.0 (Chicago, IL).

## SUPPLEMENTARY DATA FIGURES AND TABLES




