# Supplementary material for: HBV polymerase overexpression due to large core gene deletion enhances hepatoma cell growth by binding inhibition of microRNA-100
Source: Oncotarget. 2016 Jan 25;7(8):9448–61. doi: 10.18632/oncotarget.7021 (PMC4891051; doi:10.18632/oncotarget.7021)
Supplement: Supplementary file 1 [file oncotarget-07-9448-s001.pdf]

SUPPLEMENTARY DATA

SUPPLEMENTARY RESULTS

LFCD mutants driven by native HBV promoter/enhancer also led to over-expression of polymerase protein as well as enhanced hepatoma cell growth

To eliminate the possibility that HBV preC-pol overexpression in LFCD was merely due to a potent CMV promoter but not due to deletion of core protein, the CMV promoter was removed from pRc/CMV vector to generate a plasmid containing 1.3-unit length HBV genome with transcription of all RNA molecules under HBV promoters and enhancers (pHBV1.3). In addition, 4 different LFCDs from Figure 1B were introduced into the corresponding regions in pHBV1.3 to generate pHBV1.3-476 (patient –4), pHBV1.3-477 (patient –11), pHBV1.3-150 (patient –9) and pHBV1.3-510 (patient –15) plasmids, respectively. Subcellular localization and expression levels of polymerase or pol-N-mut in HepG2 cells transfected with pHBV1.3 or pHBV1.3 carrying the 4 LFCDs were assessed. Similar to those in CMV promoter driven plasmids (Figure 1C and S1A), the expression level of preC-pol in HepG2 cells transfected with pHBV1.3-477 was higher than that with pHBV1.3 (Figures S3A and S3B). The expression levels of polymerase in the other three types of LFCD were also greater than the pHBV1.3 (Figures S3A and S3B). When analyzed by immunoblot following immunoprecipitation with HBV polymerase antibody, increased polymerase expression level was also observed in HepG2 cells transfected with pHBV1.3-476, -477 and -510 (Figure S3C). Five HepG2 stable cell lines transfected by pHBV1.3, pHBV1.3-476, pHBV1.3-477, pHBV1.3-150 or pHBV1.3-510 were generated for xenograft tumor formation. Sixteen mice were divided into 4 groups for subcutaneously injecting with HepG2 cells stably transfected with the 4 LFCD mutants on the right side of back. All mice were injected with HepG2 cells stably transfected with pHBV1.3 on the corresponding left side of back for comparison. The average tumor weight increased in all groups expressing LFCD, when compared with pHBV1.3 controls (right panel) (Figure S3D). Among the 4 groups, the xenografts carrying pHBV1.3-150 and pHBV1.3-510 showed statistically significant increase of tumor weight (P = 0.029 for both).

SUPPLEMENTARY METHODS

Construction of plasmids containing HBV genome with LFCDs driven by native HBV promoters/enhancers and generation of stable cell lines

To generate a plasmid containing 1.3-unit length of HBV genome, site directed mutagenesis experiments were performed. Firstly, PCR was performed using two mismatched primers, P1040, 5’-GTGGA**G**ATCTTGCCTTAATGCCTTTG-3’ (nt. 1040 to 1065, sense; the mismatch nucleotide [underlined] was used to generate a *Bgl*II site) and P2437, 5’-AGATCTTCTGCGACGCGGCGATTG**G**GATC-3’ (nt. 2437 to 2409, antisense; the mismatched nucleotide [underlined] was used to eliminate the 3’ *Bgl*II site in order to utilize the 5’ *Bgl*II site). The plasmid, pCMV-HBV, was used as the PCR template to obtain a DNA fragment (nt. 1040 to 2437) flanked by two *Bgl*II sites. Subsequently, the *Bgl*II (nt. 12 on pRC/CMV vector) to *Bgl*II (nt. 2432 on HBV genome) DNA fragment in pCMV-HBV, which contained the CMV promoter, was replaced by the PCR-derived HBV-DNA fragment. The final construct, pHBV1.3, contained a 1.3-unit length HBV genome and the CMV promoter was deleted.

To construct plasmids with LFCD mutants driven by the native HBV promoter/enhancer, two complementary primers (30 bases each, forward and reverse) completely matched to the deletion regions (Figure 1, No. 4, 11, and 15) were synthesized. Two amplicons, one derived from the complementary reverse primer to P1040 primer and the other derived from the complementary forward primer to P2437 primer, were mixed together after gel-purification for a second stage PCR using P1040 and P2437 primers. The resulting fragments containing the deletions were used to replace the corresponding *Bgl*II to *Bgl*II region in pHBV1.3. The final products were sequence-verified and named pHBV1.3-476, pHBV1.3-477, and pHBV-510, respectively.

Finally, to construct the plasmid expressing the mutant derived from patient No. 9, two complementary primers completely matched to the deleted region were used together with P1040 and a further downstream primer, 5’-CCAAGAATATGGTGACCCGC-3’ (nt. 2840 to 2821, antisense; containing a *BstE*II site) to perform PCR-based site-directed mutagenesis as described in the

last paragraph. The PCR product containing the deletion was used to replace the *Bgl*II to *BstE*II region in pHBV1.3. The final plasmid was sequence-verified and named pHBV1.3-150.

Cell culture and stable cell line generation

To generate cells stably expressed plasmids containing native HBV promoters/enhancers, but not CMV promoter. HepG2 cells were stably transfected with pHBV1.3, pHBV1.3-476, pHBV1.3-477, pHBV1.3-150 and pHBV1.3-510 plasmids using jetPRIME reagent (Polyplus-transfection SA, Illkirch, France). The stable transformants were selected under 1 mg/mL of G418. The culture medium of transformants was collected for HBsAg measurement to confirm the success of stable transfection.

Xenograft model for HepG2 cells with knockdown of miR-100

The pmiRZip-100 and pmiRZip were transduced into HepG2 cells using lentivector expression system. The infected HepG2 cells then subcutaneously injected into four-week-old mice for observation of tumor formation.

Annexin V and PI staining by flow cytometry

Cells were treated with or without TRAIL, and then stained with fluorescein isothiocyanate (FITC)-conjugated annexin V and propidium iodide (PI) for 15 min at room temperature (Annexin V:FITC Apoptosis Detection Kit; BD Pharmingen, CA, USA). After incubation, the cell fractionation data were obtained from flow cytometer (BD FACSCalibur) and analyzed using CellQuest software.

SUPPLEMENTARY NOTE

The sequencing results of HBV genome (nt 1814-2542) from 5 HCC patients (patient– 4, 9, 11, 15, 16).

Patient–4

ATG CAA CTT TTT CAA CTC CCG CTT ACA GAC CAC CAA **ATG** CCC CTA TCT TAT CAA CAC TTC CGG AAA CTA CTG TTG TTA GAC GAC GAG GCA GGT CCC CTA GAA GAA GAA CTC CCT CGC CTT GCA GAC GAA GGT CTC AAT CGC CGC GTC GCA GAA GAT CTC AAT CTC GGG AAT CTC AAT GTT AGT ATC CCT TGG ACG CAT AA GGT GGG AAA CTT TAC TGG GCT TTA TTC TTC TAC TGT ACC TGT CTT TAA TCC TGA ATG GCA AAC TCC TTC CTT

HBV genome nt 1814-2542 vs. No.4

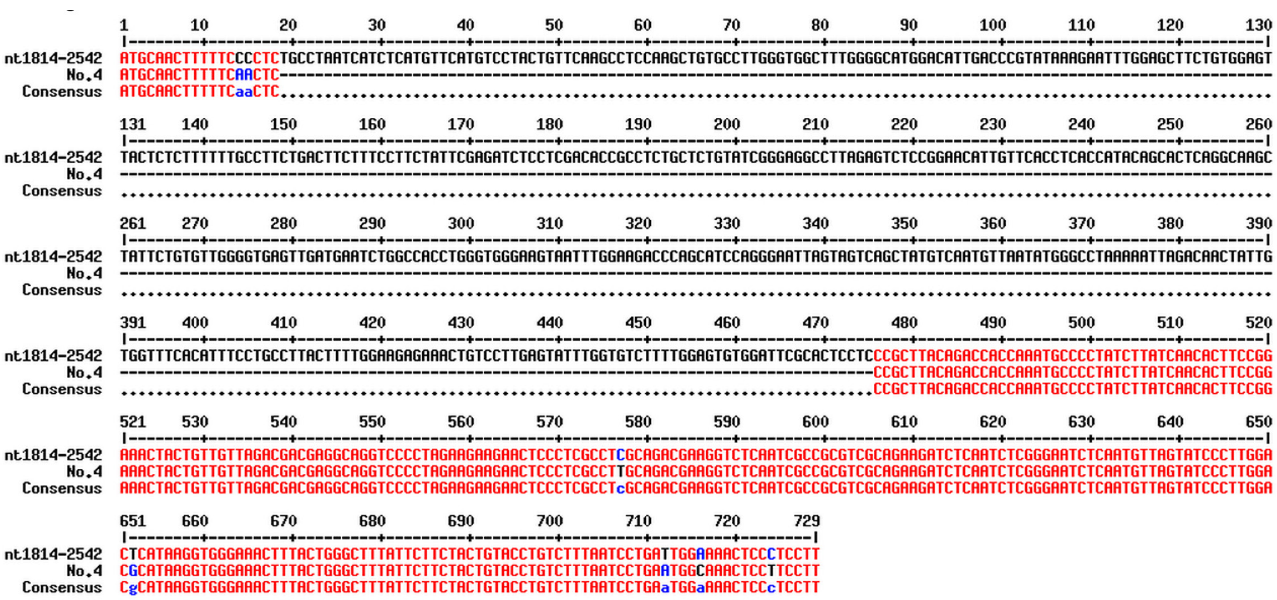

Patient–9

ATG CAA CTT TTT CAC CTC TCC CTT GGA CGC ATA AGG TGG GAA ACT TTA CTG GGC TTT ATT CTT CTA CTG TAC CTG TCT TTA ATC CTG AAT GGC AAA CTC CTT CCT T

HBV genome nt 1814-2542 vs. No.9

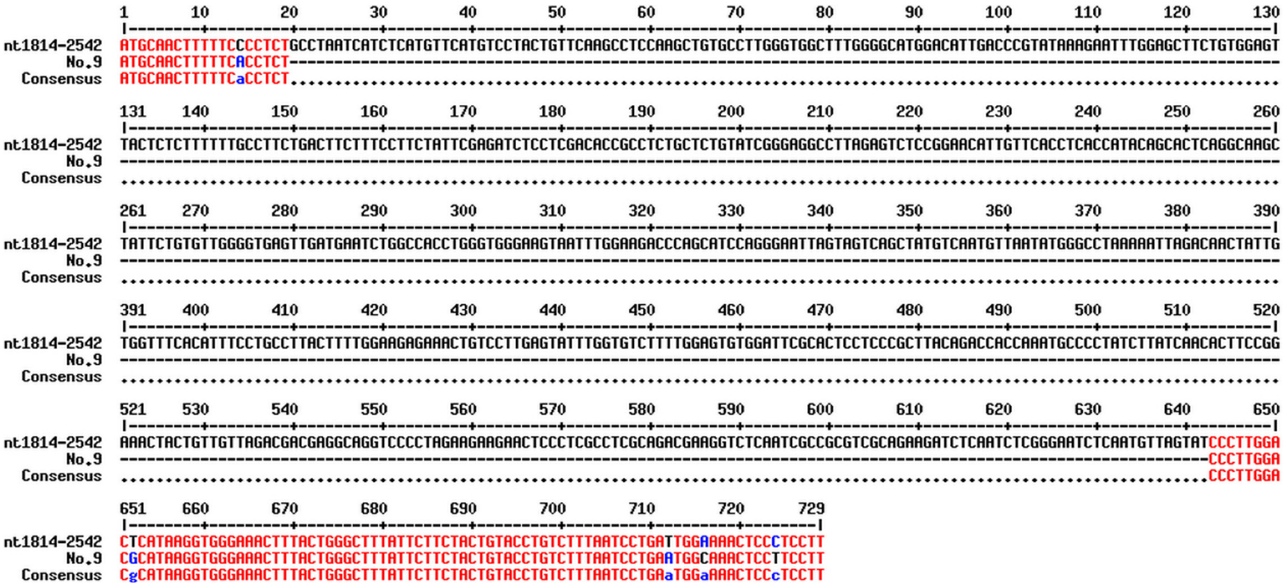

Patient–11

ATG CAA CTT TTT CAC CTC TCG CTT ACA GAC CAC CAA **ATG** CCC CTA TCT TAT CAA CAC TTC CGG AAA CTA CTG TTG TTA GAC GAC GAG GCA GGT CCC CTA GAA GAA GAA CTC CCT CGC CTC GCA GAC GAA GGT CTC AAT CGC CGC GTC GCA GAA GAT CTC AAT CTC GGG AAT CTC AAT GTT AGT ATC CCT TGG ACG CAT AAG GTG GGA AAC TTT ACT GGG CTT TAT TCT TCT ACT GTA CCT GTC TTT AAT CCT GAA TGG CAA ACT CCC TCC TT

HBV genome nt 1814-2542 vs. No.11

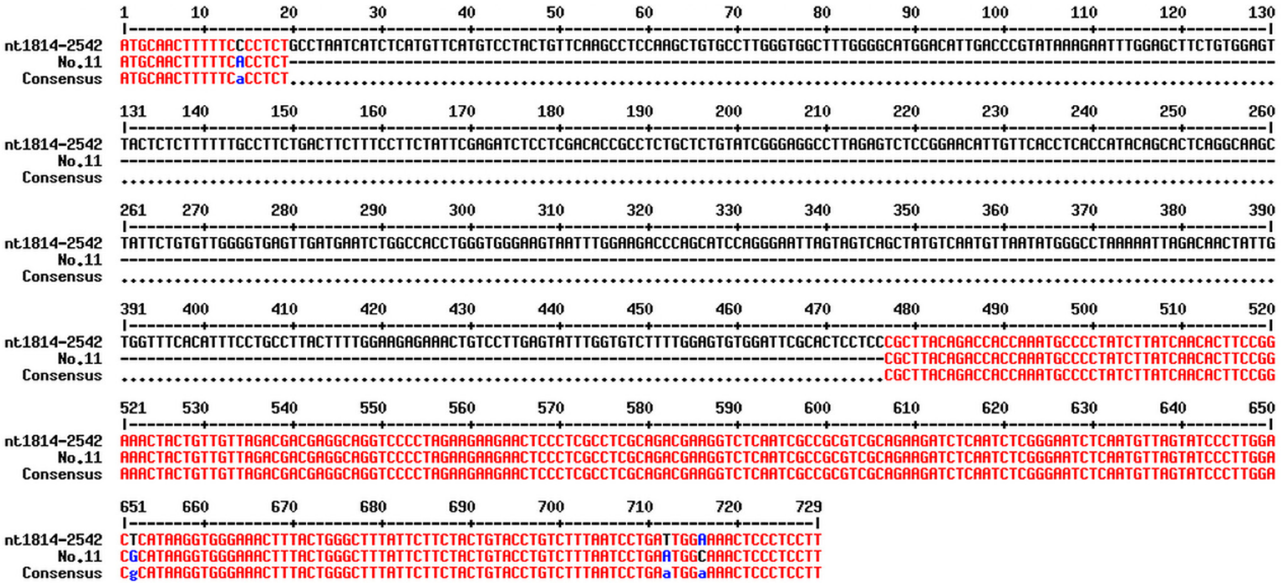

Patient–15

ATG CAA CTT TTT CAA CTC TTC CGG AAA CTA CTG TTG TTA GAC GAC GAG GCA GGT CCC CTA GAA GAA GAA CTC CCT CGC CTC GCA GAC GAA GGT CTC AAT CGC CGC GTC GCA GAA GAT CTC AAT CTC GGG GAT CTC AAT GTT AGT ATC CCT TGG ACT CAT AAG GTG GGA AAC TTT ACT GGG CTT TAG TCT TCT ACT GCA CCT GTC CTT AAT CCT GAA TGG CAA ACT CCT TCC T

HBV genome nt 1814-2542 vs. No. 15

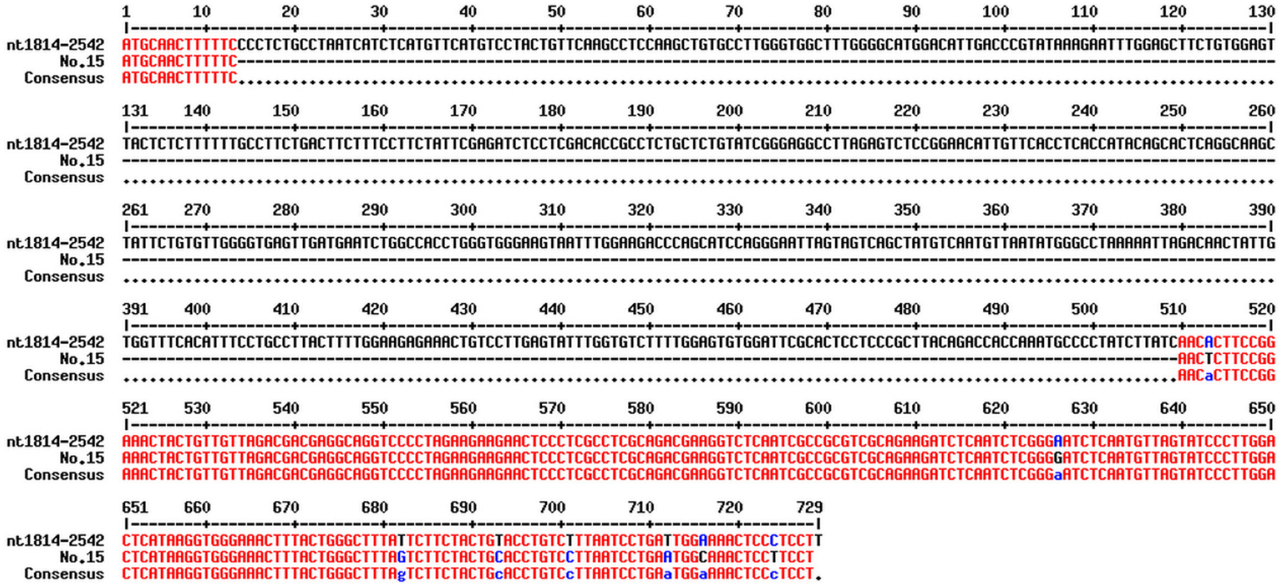

Patient–16

ATG CAA CTT TCC CCT CCT CCC GCT TAC AGA CCA CCA AAT GCC CCT ATC TTA TCA ACA CTT CCG GAA ACT ACT GTT GTT AGA CGA CGA GGC AGG TCC CCT AGA AGA AGA ACT CCC TCG CCT CGC AGA CGA AGG TCT CAA TCG CCG CGT CGC AGA AGA TCT CAA TCT CGG GAA TCT CAA TGT TAG TAT CCC TTG GAC GCA TAA GGT GGG AAA CTT TAC TGG GCT TTA TTC TTC TAC TGT ACC TGT CTT TAA TCC TGA ATG GCA AAC TCC TTC CTT

HBV genome nt 1814-2542 vs. No. 16

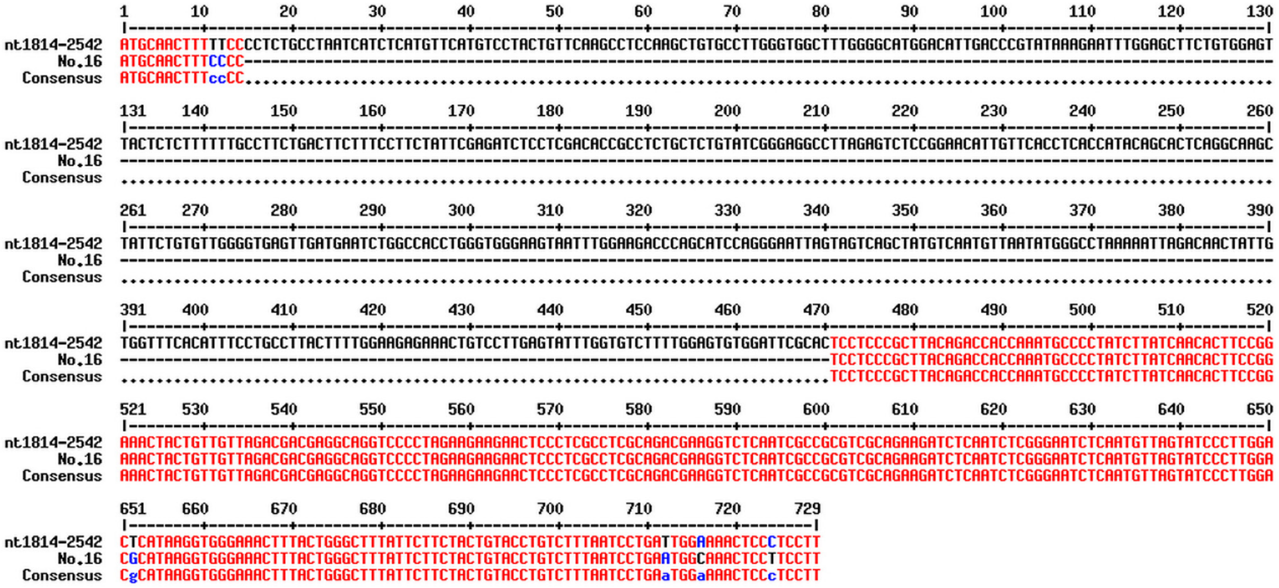

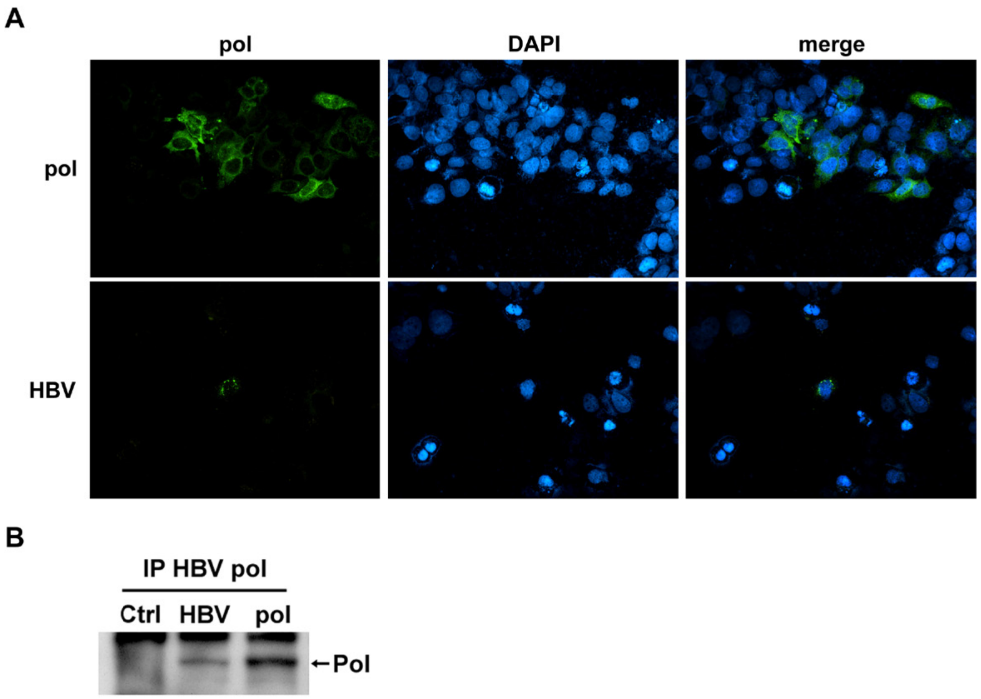

Supplementary Figure S1: Subcellular localization and protein expression of HBV polymerase or precore-polymerase fusion product in HepG2 cells. A. Immunoflouresence analysis for HBV polymerase in HepG2 cells transfected with pCMV-HBV and pCMV-pol, respectively. Nuclei were counterstained with DAPI. **B.** HepG2 cells were transfected with pRC/CMV (Ctrl), pCMV-HBV (HBV) and pCMV-pol (pol), respectively. Cell lysates were immunoprecipitated with HBV polymerase antibody followed by immunoblot analysis.

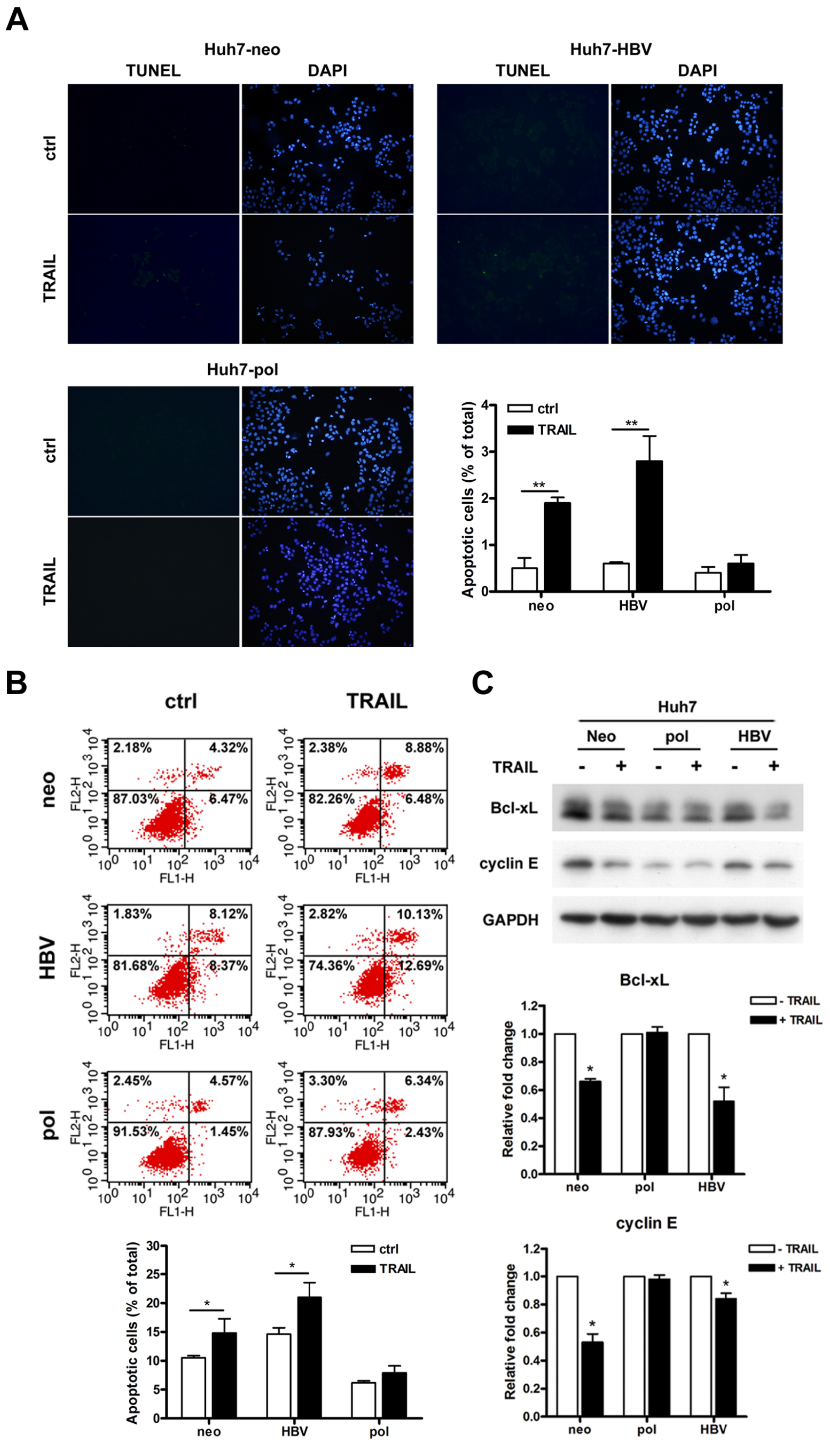

Supplementary Figure S2: HBV carrying LFCD mutation inhibited TRAIL-induced cell apoptosis in Huh7 cells. A. TUNEL assays for Huh7-neo, -pol and -HBV cells after they were treated with 100 ng/mL of TRAIL for 24 h. Cells exhibited green fluorescence were under apoptosis. Nuclei were stained with DAPI. Quantitative comparison for the numbers of apoptotic cells were depicted (right lower panel). **B.** After treated with TRAIL, Huh7 stable cells were stained with annexin V-FITC and PI for flow cytometric analysis. Lower panel, quantitative comparison of the numbers of apoptotic cells. **C.** Comparisons of Bcl-xL and cyclin E expression levels after Huh7 cells derived stable transformants (-neo, -pol, and -HBV) were treated with (+) or without (-) TRAIL. GAPDH served as a loading control. Top panel, immunoblot analysis; Middle and bottom panels, quantitative assessments. Values were given in mean ± SD from three independent experiments. "*", $P < 0.05$; "**", $P < 0.01$.

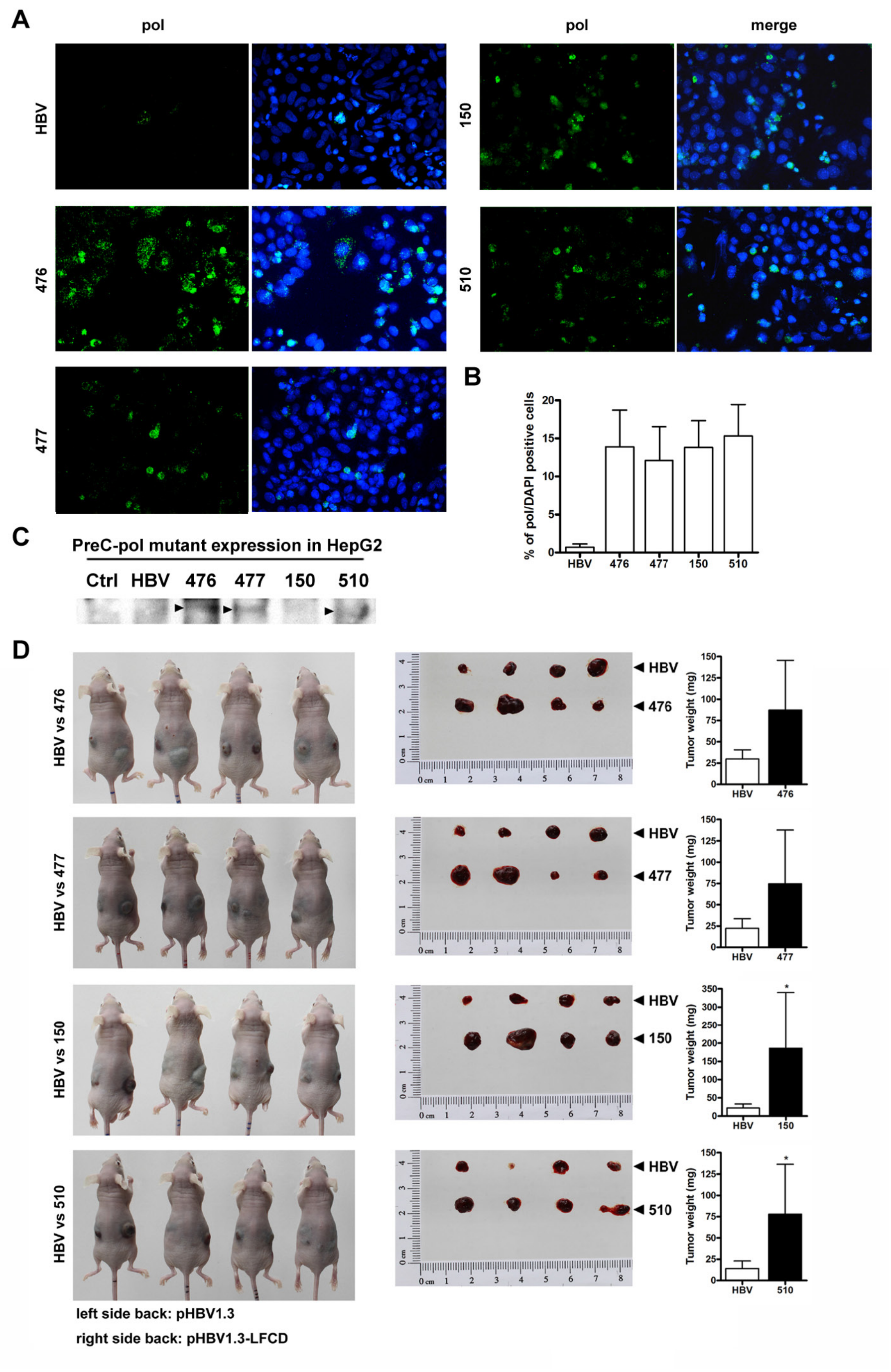

Supplementary Figure S3: Over-expression and tumorigenicity of HBV preC-pol derived from four LFCD mutants driven by native HBV promoters/enhancers. **A.** Immunoflouresence analysis for HBV polymerase in HepG2 cells transiently transfected with pHBV1.3, pHBV1.3-476, pHBV1.3-477, pHBV1.3-150 or pHBV1.3-510. Nuclei were counterstained with DAPI. **B.** Quantification of the percentages of HBV polymerase antigen positive cells. **C.** HBV polymerase proteins, wild type or core-polymerase fusion, were detected by immunoprecipitaion using anti-HBV polymerase (HBV pol) antibody followed by immunoblot. Arrowhead indicated the positions of HBV polymerase signals. **D.** Comparison of tumorigenicity in nude mice using HepG2 derived stable transformants. Cells carrying pHBV1.3-476, -477, -150 and -510 were subcutaneously injected into the right side of back, while cells carrying pHBV1.3 were injected into the left side of back for comparison (left panels). Sizes of xenograft tumors in each group were shown (middle panels). Effects of pHBV1.3 carrying the 4 LFCDs on tumor growth were demonstrated by tumor weights (right panels). The difference was examined by Mann-Whitney U test. *, $P < 0.05$.

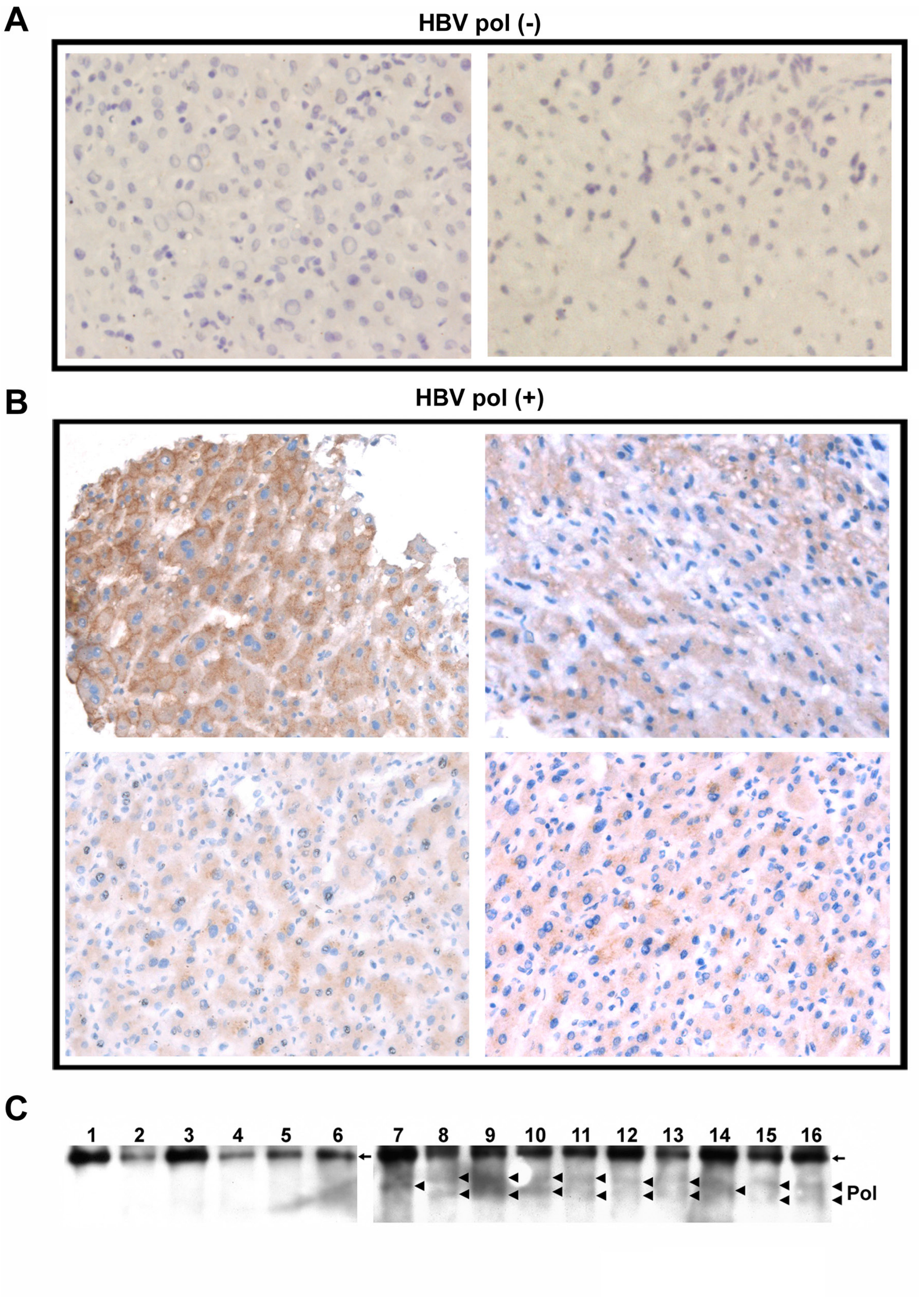

Supplementary Figure S4: Expression of HBV polymerase antigen in non-cancerous liver tissues from HCC patients. Representative results of immunohistochemical analysis showing liver tissues negative **A.** or positive **B.** for HBV polymerase antigen. The positive signals were mostly located in the cytosol as peri-nuclear patches or granules. Occasionally, a submembranous distribution could be seen (left upper panel in B). **C.** HBV polymerase antigen reacting proteins detected by immunoblot analysis following immunoprecipitation. Small arrow, non-specific background bands; Solid triangles, positions of the polymerase antigen reacting proteins. Lanes 1-6, liver tissues negative for HBV polymerase staining by immunohistochemistry. Lanes 7-16, liver tissues positive for HBV polymerase staining.

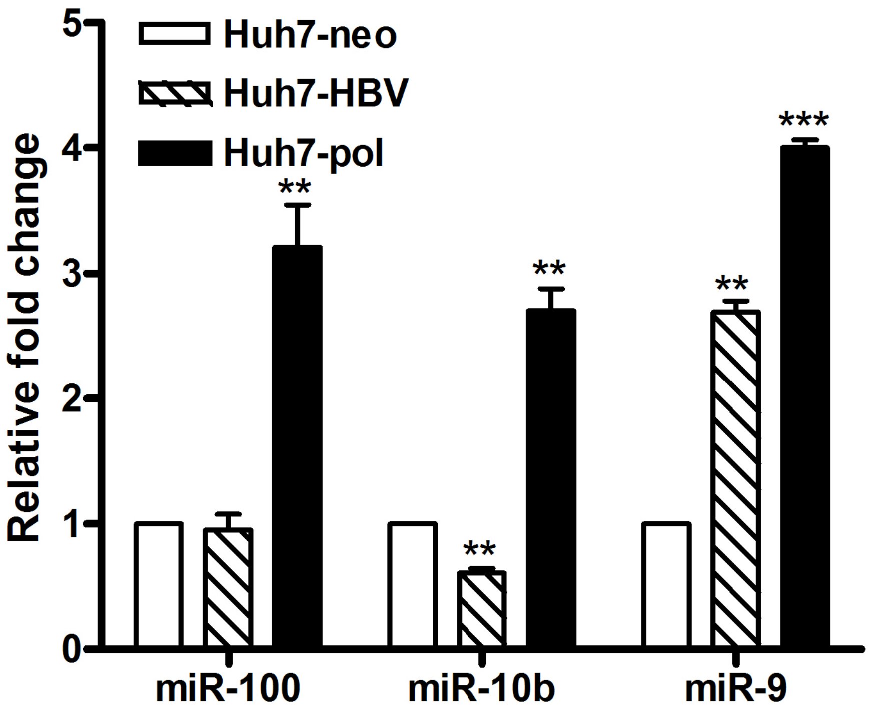

Supplementary Figure S5: Physical interaction between preC-pol and miRNAs. Huh7-neo, -HBV, and -pol stable cell lines were immunoprecipitated using HBV pol antibody. Total RNA was extracted from the immunoprecipitates and submitted for quantitative assessment for miR-100, miR-10b and miR-9 by real-time RT-qPCR. Data was from three independent experiments. "**", $P < 0.01$, "***", $P < 0.001$.

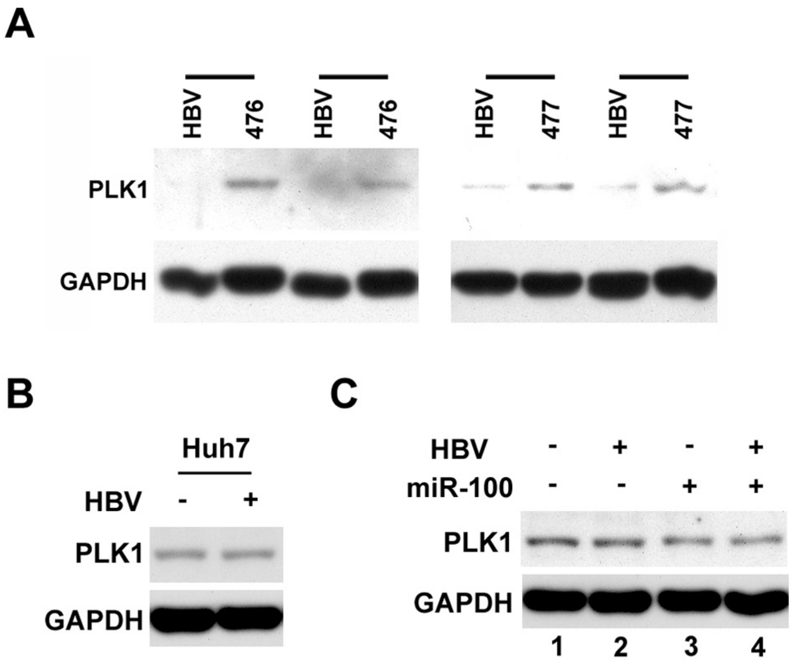

Supplementary Figure S6: Expression of PLK1 by immunoblot analysis. A. The expression levels of PLK1 in HepG2 xenograft tumors carrying pHBV1.3, pHBV1.3-476, or pHBV1.3-477. **B.** PLK1 protein levels in Huh7 cells transfected with (+) or without (-) pCMV-HBV. **C.** PLK1 protein levels in pCMV-HBV transfected or untransfected Huh7 cells with or without overexpression of miR-100. GAPDH served as a loading control.

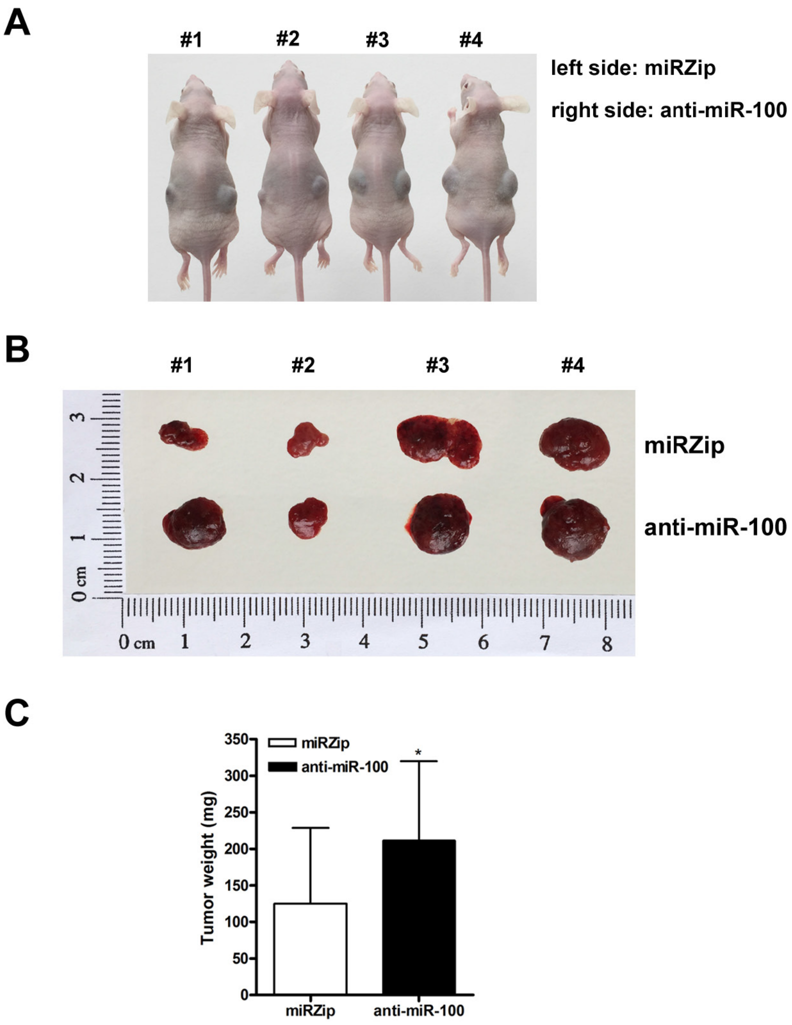

Supplementary Figure S7: Promotion of tumor growth by knockdown of miR-100 in HepG2 xenografts. A. Representative results of subcutaneously injected HepG2 cells with (anti-miR-100) or without (miRZip) miR-100 knockdown in nude mice. **B.** The dissected xenograft tumors. **C.** The calculated tumor weight. Values were mean ± SD. "*", $P < 0.05$.

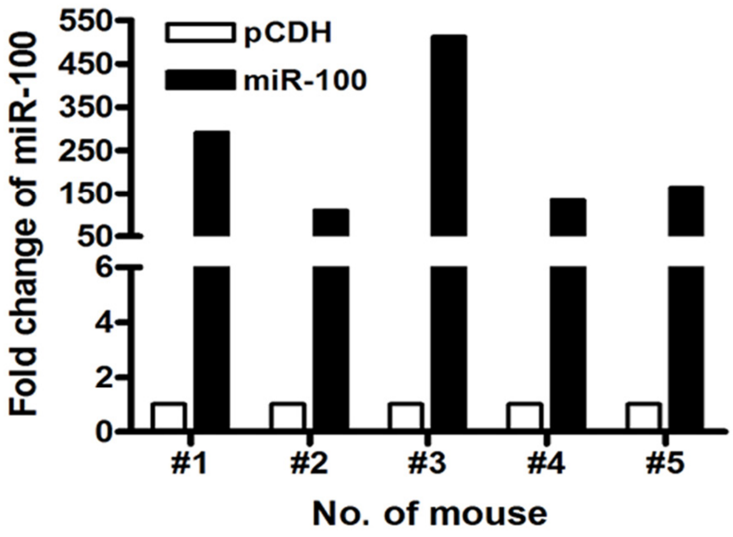

Supplementary Figure S8: Expression levels of miR-100 in Huh7-pol xenograft tumors. Paired Huh7-pol xenografts transduced with pCDH (mock) or pCDH-miR-100 derived lentiviruses for exogenous miR-100 overexpression were submitted for miR-100 quantitative assays using stem-loop RT-qPCR. The relative abundance of miR-100 was calculated.

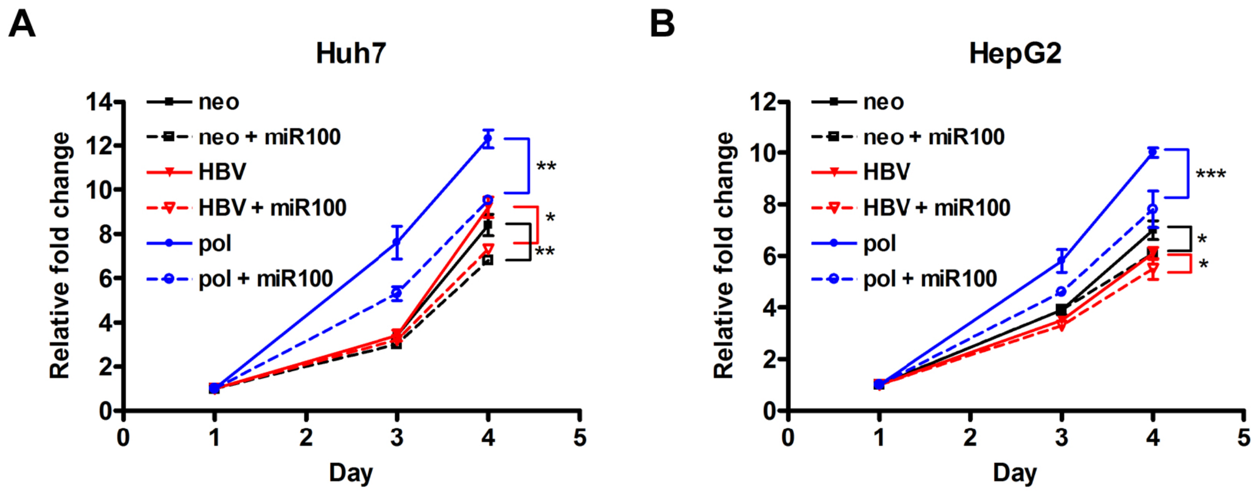

Supplementary Figure S9: Suppression of cell proliferation by miR-100. Cells proliferation rates assessed by MTT assays for Huh7-neo, HBV, and -pol cells **A.** and for HepG2-neo, -HBV, and -pol cells **B.** infected with or without miR-100. "*", $P < 0.05$; "**", $P < 0.01$; "***", $P < 0.001$.

Supplementary Table S1: Basic clinical and virological data in the case-control study to identify core mutants

Groups	Chronic hepatitis	Cirrhosis	HCC	P value[c]
Number of patients	33	33	33	
Male	100%	100%	100%	
Age (years)[a]	54.8 ± 11.3	55.3 ± 11.4	55.3 ± 11.9	0.683
Precore G1896A	21 (63.6%)	26 (78.8%)	24 (72.7%)	0.388
BCP A1762T/G1764A	17 (51.5%)	19 (57.6%)	25 (75.8%)	0.109
BCP G1719T	3 (9.1%)	3 (9.1%)	9 (27.3%)	**0.034**[e]
BCP C1730G	14 (42.4%)	8 (24.2%)	18 (54.5%)	**0.041**
BCP C1799G	13 (39.4%)	12 (36.4%)	17 (51.5%)	0.419
HBeAg positive	2 (6.1%)	4 (12.1%)	2 (6.1%)	0.715[e]
Large-fragment core gene deletion	0 (0.0%)	0 (0.0%)	5 (15.2%)	**0.003**[e]
Genotype				
A	2 (6.1%)	1 (3.0%)	3 (9.1%)	0.397[e]
B	22 (66.7%)	25 (75.8%)	10 (30.3%)	**< 0.001**
C	9 (27.3%)	7 (21.2%)	20 (60.6%)	**0.002**
HBV DNA ($\times 10^6$copies/mL)[b]	0.523 (0.037 – 2534.23)	0.023 (0 – 322.33)	0.035 (0 – 237.35)	**0.011**
AST (U/L)	236.9 ± 308.7	95.4 ± 168.5	141.0 ± 130.7	**0.036**[d]
ALT (U/L)	381.6 ± 362.6	95.8 ± 199.2	74.0 ± 69.4	**< 0.001**[d]
Bilirubin (mg/dL)	1.2 ± 1.2	3.1 ± 8.3	5.9 ± 7.1	**0.022**[d]
AFP (ng/mL)[b]	9.5 ± 18.3	9.5 ± 12.5	983.7 ± 2161.9	**0.003**[d]

[a]Mean ± Standard Deviation
[b]Median (Range); The lower quantification limit for HBV DNA was 116 copies/mL; The lower quantification limit for AFP was 3 ng/mL.
[c]One-way ANOVA for parametric data and Chi-Square for dichotomous data.
ND, not done
[d]Post hoc analysis under bonferroni's correction showed significant differences between chronic hepatitis and cirrhosis groups in AST (P = 0.033) and ALT (P < 0.001), respectively; between chronic hepatitis and HCC groups in ALT (P < 0.001), bilirubin (P = 0.020) and AFP (P = 0.012), respectively; and between cirrhosis and HCC groups in AFP (P = 0.009). HBV-DNA level was significantly higher in chronic hepatitis group (hepatitis versus cirrhosis, P = 0.003; hepatitis versus HCC, P = 0.004).
[e]Chronic hepatitis and cirrhosis groups combined.

Supplementary Table S2: Core gene substitution mutations in patients with chronic hepatitis, cirrhosis and HCC

Variation site No.	Core gene mutation[a]	Chronic hepatits	Cirrhosis	HCC	*P* value[b]	Genotype association[d]
		n = 33	n = 33	n = 33		
1	G1915A(T)(C)	6 (18.2%)	10 (30.3%)	16 (48.5%)	0.030	non-related
2	T1933A(C)	6 (18.2%)	5 (15.2%)	20 (60.6%)	< 0.001	non-related
3	T1936C(A)(G)	0 (0.0%)	6 (18.2%)	3 (9.1%)	0.037	non-related
4	T1938C(G)(A)	9 (27.3%)	2 (6.1%)	3 (9.1%)	0.028	non-related
5	G2027A(T)	0 (0.0%)	0 (0.0%)	3 (9.1%)	0.045	non-related
6	A2059G(T)	19 (57.6%)	19 (57.6%)	4 (12.1%)	< 0.001	B
7	C2063A(T)	8 (24.2%)	3 (9.1%)	1 (3.0%)	0.025	non-related
8	G2104A	18 (54.5%)	19 (57.6%)	4 (12.1%)	< 0.001	A, B
9	A2120G	7 (21.2%)	8 (24.2%)	1 (3.0%)	0.041	B
10	C2134T(G)	19 (57.6%)	21 (63.6%)	6 (18.2%)	< 0.001	A, B
11	C2136A	7 (21.2%)	1 (3.0%)[c]	2 (6.1%)	0.037[c]	non-related
12	A2144C	3 (9.1%)	0 (0.0%)[c]	0 (0.0%)	0.050[c]	C2
13	C2158A	3 (9.1%)	4 (12.9%)[c]	17 (51.5%)	< 0.001[c]	non-related
14	C2183T(A)	9 (27.3%)	2 (6.5%)[c]	1 (3.0%)	0.005[c]	non-related
15	A2233G	16 (48.5%)	11 (35.5%)[c]	2 (6.1%)	0.001[c]	B
16	A2239C	9 (27.3%)	4 (12.9%)[c]	1 (3.0%)	0.019[c]	non-related
17	G2251A	24 (72.7%)	19 (61.3%)[c]	4 (12.1%)	< 0.001[c]	B
18	G2260A(C)	10 (30.3%)	11 (33.3%)	23 (69.7%)	0.002	non-related
19	T2263G	1 (3.0%)	2 (6.1%)	10 (30.3%)	0.002	non-related
20	C2290T(A)(G)	22 (66.7%)	23 (69.7%)	8 (24.2%)	< 0.001	B
21	T2293A(C)	18 (54.5%)	19 (57.6%)	4 (12.1%)	< 0.001	B
22	C2296T	19 (57.6%)	20 (60.6%)	4 (12.1%)	< 0.001	A, B
23	C2304A	14 (42.4%)	12 (36.4%)	1 (3.0%)	< 0.001	B4
24	G2352A(C)	9 (27.3%)	5 (15.2%)	1 (3.0%)	0.023	non-related
25	C2354A(G)	14 (42.4%)	19 (57.6%)	4 (12.1%)	0.001	B
26	C2366A	3 (9.1%)	0 (0.0%)	0 (0.0%)	0.045	non-related

[a]All nucleotide substitutions found in the three groups were listed. The substituted nucleotides listed behind the nucleotide numbers were arranged according to the frequency from high to low.
[b]Chi-Square analysis for all groups.
[c]Two patients with cirrhosis have CID located at nt. 2136 to 2258. Thus, only 31 cirrhotic patients were included for statistical analysis.
[d]Genotype association for the first nucleotide substitution listed immediately behind the number of nucleotide.

Supplementary Table S3: Baseline characteristics of 179 HBV-related HCC patients

Characteristics	Total (n=179)	Genotype B (n=117)	Genotype C (n=62)	P value[c]
Age (years) [a]	51.01 ± 13.62	50.14 ± 14.00	52.65 ± 12.87	0.231
Gender, Male, n (%)	155 (86.6%)	97 (82.9%)	58 (93.5%)	**0.047**
Cirrhosis, n (%)	101 (56.4%)	59 (50.4%)	42 (67.7%)	**0.026**
Alcoholism, n (%)	32 (17.9%)	20 (17.1%)	12 (19.4%)	0.707
Ascites, n (%)	18 (10.1%)	13 (11.1%)	5 (8.1%)	0.519
**HBV status**				
HBV-DNA (copies/gram)	22.67 (0-22455.5)	20.56 (0-22455.5)	28.99 (0-5551.38)	0.096
Basal core promoter, A1762T/G1764A, n (%)	104 (58.1%)	52 (44.4%)	52 (83.9%)	**<0.001**
Precore stop codon, G1896A, n (%)	119 (66.5%)	87 (74.4%)	32 (51.6%)	**0.002**
Total Pre-S deletion mutation, n (%)	55 (30.7%)	21 (17.9%)	34 (54.8%)	**<0.001**
Large fragment Pre-S deletion mutation, n (%)	37 (20.7%)	15 (12.8%)	22 (35.5%)	**<0.001**
Small fragment Pre-S deletion mutation, n (%)	20 (11.2%)	6 (5.1%)	14 (22.6%)	**<0.001**
Large fragment core deletion mutation, n (%)	24 (13.4%)	13 (11.1%)	11 (17.7%)	0.215
**Tumor status**				
Grade (1/2/3/4)	6/49/109/15	4/34/70/9	2/15/39/6	0.894
Size (cm) [a]	7.34 ± 7.08	7.24 ± 5.25	7.52 ± 9.69	0.830
Tumor number (1/2/3/4)	85/55/31/8	58/34/20/5	27/21/11/3	0.884
Capsule, n (%)	129 (72.1%)	84 (71.8%)	45 (72.6%)	0.387
Macrovascular invasion, n (%)	33 (18.4%)	22 (18.8%)	11 (17.7%)	0.862
Microvascular invasion, n (%)	64 (35.8%)	43 (36.8%)	21 (33.9%)	0.702
**Biochemistry**				
Total bilirubin (mg/dL) [a]	1.37 ± 1.74	1.44 ± 2.03	1.23 ± 0.97	0.349
AST(U/L) [a]	85.97 ± 116.93	85.23 ± 122.30	87.37 ± 107.03	0.904
ALT(U/L) [a]	79.98 ± 107.61	70.37 ± 85.48	98.11 ± 139.21	0.156
Albumin (g/dL) [a]	3.85 ± 0.63	3.83 ± 0.66	3.88 ± 0.58	0.646
Creatinine (mg/dL) [a]	1.23 ± 1.34	1.31 ± 1.65	1.08 ± 0.24	0.139
Prothrombin time (sec) [a]	12.97 ± 8.14	12.19 ± 1.53	14.42 ± 13.59	0.083
Alpha-fetoprotein (ng/mL) [b]	30.5 (1.47-327500)	20 (1.47-327500)	36 (2-45164)	0.723

[a] Mean ± Standard Deviation
[b] Median (Range); The lower quantification limit for AFP was 3 ng/mL.
[c] Student t test for continuous variables with normal distribution, Mann-Whitney's U test was used for continuous variables with a non-normal distribution, and Chi-Square for dichotomous data.

Supplementary Table S4: Cox proportional hazard model analysis of clinical and virological factors associated with postoperative recurrence-free and overall survivals

See Supplementary File 1
