# Supplementary material for: HBV polymerase overexpression due to large core gene deletion enhances hepatoma cell growth by binding inhibition of microRNA-100
Source: Oncotarget. 2016 Jan 25;7(8):9448–61. doi: 10.18632/oncotarget.7021 (PMC4891051; doi:10.18632/oncotarget.7021)
Supplement: Supplementary file 2 [file oncotarget-07-9448-s002.doc]

**Table S4. Cox proportional hazard model analysis of clinical and virological factors associated with postoperative recurrence-free and overall survivals**

|  | | |  | **Recurrence-free survival** | | | | |  | **Overall Survival** | | | | |
| --- | --- | --- | --- | --- | --- | --- | --- | --- | --- | --- | --- | --- | --- | --- |
|  | |  |  | **Univariate Analysis** |  | **Multivariate Analysis** | | |  | **Univariate Analysis** |  | **Multivariate Analysis** | | |
| **Parameters** | | **n** |  | **P value^b^** |  | **HR** | **95% CI** | **P value^c^** |  | **P value^b^** |  | **HR** | **95% CI** | **P value^c^** |
| Ages (years) ^a^ | ≤ 50 | 89 |  |  |  |  |  |  |  |  |  |  |  |  |
|  | >50 | 90 |  | 0.132 |  |  |  |  |  | 0.065 |  |  |  |  |
| Gender | Female | 24 |  |  |  |  |  |  |  |  |  |  |  |  |
|  | Male | 155 |  | 0.426 |  |  |  |  |  | 0.401 |  |  |  |  |
| Cirrhosis | No | 78 |  |  |  |  |  |  |  |  |  |  |  |  |
|  | Yes | 101 |  | 0.360 |  |  |  |  |  | 0.750 |  |  |  |  |
| Alcoholism | No | 147 |  |  |  |  |  |  |  |  |  |  |  |  |
|  | Yes | 32 |  | 0.748 |  |  |  |  |  | 0.704 |  |  |  |  |
| Ascites | No | 161 |  |  |  |  |  |  |  |  |  |  |  |  |
|  | Yes | 18 |  | **0.002** |  | 2.057 | 1.109 – 3.817 | **0.022** |  | **0.008** |  | 2.914 | 1.109 – 7.661 | **0.030** |
| **HBV status** |  |  |  |  |  |  |  |  |  |  |  |  |  |  |
| HBV genotype | B | 117 |  |  |  |  |  |  |  |  |  |  |  |  |
|  | C | 62 |  | **0.013** |  | 1.328 | 0.816 – 2.162 | 0.254 |  | 0.740 |  |  |  |  |
| HBV-DNA (copies/gram) ^a^ | ≤ 22.67 | 89 |  |  |  |  |  |  |  |  |  |  |  |  |
|  | >22.67 | 90 |  | **0.007** |  | 1.343 | 0.877 – 2.056 | 0.175 |  | 0.081 |  |  |  |  |
| Basal core promoter, A1762T/G1764A | No | 74 |  |  |  |  |  |  |  |  |  |  |  |  |
|  | Yes | 105 |  | **0.001** |  | 1.670 | 1.025 – 2.721 | **0.039** |  | **0.023** |  | 2.938 | 1.095 – 7.883 | **0.032** |
| Precore stop codon, G1896A | No | 60 |  |  |  |  |  |  |  |  |  |  |  |  |
|  | Yes | 119 |  | 0.750 |  |  |  |  |  | 0.694 |  |  |  |  |
| Total Pre-S deletion mutation | No | 124 |  |  |  |  |  |  |  |  |  |  |  |  |
|  | Yes | 55 |  | 0.209 |  |  |  |  |  | 0.553 |  |  |  |  |
| Large fragment Pre-S deletion mutation | No | 142 |  |  |  |  |  |  |  |  |  |  |  |  |
|  | Yes | 37 |  | 0.402 |  |  |  |  |  | 0.076 |  |  |  |  |
| Small fragment Pre-S deletion mutation | No | 159 |  |  |  |  |  |  |  |  |  |  |  |  |
|  | Yes | 20 |  | **<0.001** |  | 1.262 | 0.686 – 2.323 | 0.454 |  | 0.089 |  |  |  |  |
| Large fragment core deletion mutation | No | 155 |  |  |  |  |  |  |  |  |  |  |  |  |
|  | Yes | 24 |  | **<0.001** |  | 2.073 | 1.159 – 3.709 | **0.014** |  | **0.036** |  | 1.629 | 0.629 – 4.220 | 0.315 |
| **Tumor status** |  |  |  |  |  |  |  |  |  |  |  |  |  |  |
| Grade (1/2/3/4) | 1&2 | 55 |  |  |  |  |  |  |  |  |  |  |  |  |
|  | 3&4 | 124 |  | 0.120 |  |  |  |  |  | 0.437 |  |  |  |  |
| Size ^a^ | ≤ 5.5 | 97 |  |  |  |  |  |  |  |  |  |  |  |  |
|  | > 5.5 | 82 |  | **<0.001** |  | 1.489 | 0.937 – 2.366 | 0.092 |  | **0.006** |  | 2.172 | 0.864 –5.458 | 0.099 |
| Tumor number | ≤ 2 | 140 |  |  |  |  |  |  |  |  |  |  |  |  |
|  | > 2 | 39 |  | 0.363 |  |  |  |  |  | 0.418 |  |  |  |  |
| Capsule | No | 50 |  |  |  |  |  |  |  |  |  |  |  |  |
|  | Yes | 129 |  | 0.789 |  |  |  |  |  | 0.921 |  |  |  |  |
| Macrovascular invasion | No | 146 |  |  |  |  |  |  |  |  |  |  |  |  |
|  | Yes | 33 |  | 0.537 |  |  |  |  |  | 0.298 |  |  |  |  |
| Microvascular invasion | No | 115 |  |  |  |  |  |  |  |  |  |  |  |  |
|  | Yes | 64 |  | **<0.001** |  | 1.638 | 1.023 – 2.621 | **0.040** |  | 0.324 |  |  |  |  |
| **Biochemistry** |  |  |  |  |  |  |  |  |  |  |  |  |  |  |
| Total bilirubin (mg/dL) ^a^ | ≤ 1.0 | 103 |  |  |  |  |  |  |  |  |  |  |  |  |
|  | > 1.0 | 76 |  | 0.664 |  |  |  |  |  | 0.367 |  |  |  |  |
| AST (U/L) ^a^ | ≤ 48 | 91 |  |  |  |  |  |  |  |  |  |  |  |  |
|  | > 48 | 88 |  | **<0.001** |  | 1.304 | 0.811 – 2.099 | 0.274 |  | **0.005** |  | 1.941 | 0.742 – 5.075 | 0.176 |
| ALT (U/L) ^a^ | ≤ 42 | 90 |  |  |  |  |  |  |  |  |  |  |  |  |
|  | > 42 | 89 |  | 0.273 |  |  |  |  |  | 0.867 |  |  |  |  |
| Albumin (g/dL) ^a^ | ≤ 3.9 | 96 |  |  |  |  |  |  |  |  |  |  |  |  |
|  | > 3.9 | 83 |  | 0.081 |  |  |  |  |  | 0.065 |  |  |  |  |
| Creatinine (mg/dL) ^a^ | ≤ 1.0 | 90 |  |  |  |  |  |  |  |  |  |  |  |  |
|  | > 1.0 | 89 |  | 0.839 |  |  |  |  |  | 0.511 |  |  |  |  |
| Prothrombin time (sec)^a^ | ≤ 12 | 90 |  |  |  |  |  |  |  |  |  |  |  |  |
|  | > 12 | 89 |  | **0.043** |  | 1.326 | 0.817 – 2.150 | 0.253 |  | 0.200 |  |  |  |  |
| AFP (ng/mL) ^a^ | ≤ 30.5 | 89 |  |  |  |  |  |  |  |  |  |  |  |  |
|  | > 30.5 | 90 |  | **0.003** |  | 1.529 | 0.981 – 2.382 | 0.061 |  | 0.064 |  |  |  |  |

^a^ Divided to two groups by median.

^b^ Evaluated by univariate Cox proportional hazard model.

^c^ The parameters with *P* < 0.05 in univariate analysis were further evaluated by multivariate Cox proportional hazard model.

Abbreviations: HR: hazard ratio; CI: confidence interval.
